# Targeting the SIRT1‐NAT10‐GABABR1 Axis: A Novel Epitranscriptomic Approach to Mitigate Sevoflurane‐Induced Cognitive Impairment in Aging

**DOI:** 10.1002/cns.70762

**Published:** 2026-02-04

**Authors:** Xin Xie, Wei Du, Yanmei Zhang, Xiaomin Zhang

**Affiliations:** ^1^ Department of Anesthesiology, Liaoning Cancer Hospital & Institute Cancer Hospital of China Medical University Shenyang China

**Keywords:** gamma‐aminobutyric acid type B receptor subunit 1, mRNA acetylation, N‐acetyltransferase 10, postoperative cognitive dysfunction, sevoflurane, Sirtuin 1

## Abstract

**Aims:**

This study investigated how Sirtuin 1 (Sirt1) protects against sevoflurane‐induced postoperative cognitive dysfunction (POCD) in aged rats by targeting N‐acetyltransferase 10 (NAT10)‐mediated mRNA acetylation and mitochondrial homeostasis.

**Methods:**

Aged rats received sevoflurane exposure and AAV‐mediated *Sirt1*/*Nat10* manipulation. We assessed autophagy (WB, LC3/TOM20 colocalization), energy metabolism (ROS/ATP, JC‐1), and *Gababr1* expression (RT‐qPCR, immunofluorescence). Cognitive function was evaluated using Y‐maze, NORT, and MWM. scRNA‐seq identified neuronal subpopulations, while RIP‐qPCR/dot blot analyzed *Nat10*‐*Gababr1* mRNA interactions. Patch‐clamp recordings measured IPSC_slow amplitudes.

**Results:**

Sevoflurane increased NAT10 expression and *Gababr1* mRNA ac4C acetylation. Sirt1 overexpression deacetylated NAT10, restoring autophagy (↑LC3‐II), reducing ROS, and improving cognition. scRNA‐seq revealed SIRT1 enrichment in high‐autophagy neurons. *Nat10* knockdown decreased *Gababr1* expression and cognitive deficits. Electrophysiology confirmed SIRT1‐mediated reduction of Baclofen‐induced IPSC_slow via NAT10 deacetylation.

**Conclusion:**

SIRT1 alleviates POCD by deacetylating NAT10 to reduce *Gababr1* mRNA acetylation, thereby normalizing synaptic inhibition and restoring metabolic‐autophagic balance. The SIRT1‐NAT10‐GABABR1 axis represents a novel therapeutic target for anesthesia‐related neurotoxicity.

## Graphical abstract

The study reveals that NAT10 enhances GABABR1 mRNA stability via ac4A modification, leading to excessive inhibitory synaptic activation. In aged rats, sevoflurane contributes to cognitive impairment by inhibiting Sirt1 and neuronal autophagy, which subsequently disrupts energy metabolism.

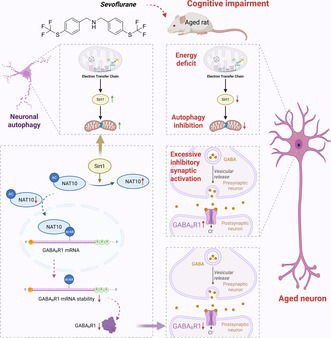

**Abbreviations**
AAV: adeno‐associated virus
ACSF: artificial cerebrospinal fluid
Ara‐C: cytosine arabinoside
ATP: adenosine triphosphate
CCK‐8: Cell Counting Kit‐8
Co‐IP: co‐immunoprecipitation
DAPI: 4′,6‐diamidino‐2‐phenylindole
DAPI: 4′,6‐diamidino‐2‐phenylindole
DEGs: differentially expressed genes
DG: dentate gyrus
GABABR1: gamma‐aminobutyric acid type B receptor subunit 1
GFP: green fluorescent protein
GO: gene ontology
hSyn: human synapsin promoter
IPSCs: inhibitory postsynaptic currents
JC‐1: 5,5′,6,6′‐tetrachloro‐1,1′,3,3′‐tetraethylbenzimidazolylcarbocyanine Iodide
KEGG: Kyoto Encyclopedia of Genes and Genomes
MMP: mitochondrial membrane potential
MWM: Morris Water Maze
NAT10: N‐acetyltransferase 10
NORT: novel object recognition test
OFT: open field test
PBS: phosphate‐buffered saline
PCA: principal component analysis
POCD: postoperative cognitive dysfunction
PVDF: polyvinylidene difluoride
RIP‐qPCR: RNA immunoprecipitation followed by quantitative polymerase chain reaction
ROS: reactive oxygen species
RT‐qPCR: real‐time quantitative polymerase chain reaction
scRNA‐seq: single‐cell RNA sequencing
Sirt1: Sirtuin 1
SPF: specific‐pathogen‐free
WB: Western blot

## 1 Introduction

Postoperative cognitive dysfunction (POCD) is a common neurological complication in elderly patients following general anesthesia, characterized by varying degrees of impairment in memory, learning ability, attention, and executive function [1, 2]. With the global trend of population aging and the increasing number of elderly individuals undergoing surgery, the incidence and clinical burden of POCD is escalating, posing a significant challenge in both anesthesiology and neuroscience [3, 4].

Growing evidence implicates impaired autophagy [5] and sevoflurane‐induced mitochondrial dysfunction [6, 7] in POCD pathogenesis, while emerging epitranscriptomic research highlights NAT10‐mediated *ac4C* mRNA modification [8, 9] as a novel regulatory layer in neurocognitive function, though its role in anesthesia‐related neurotoxicity remains unexplored.

SIRT1, a key regulator of neuroprotection and metabolism [10], can modulate NAT10 activity through deacetylation, while emerging evidence suggests the SIRT1‐NAT10 axis may influence POCD via *ac4C* mRNA modification and GABAergic synaptic regulation [11]. This study investigates whether SIRT1‐mediated NAT10 deacetylation alleviates sevoflurane‐induced POCD through *ac4C*‐dependent GABABR1 regulation, providing novel mechanistic insights into anesthesia‐related cognitive dysfunction.

In summary, this study aims to elucidate the mechanistic role of SIRT1 in alleviating sevoflurane‐induced POCD by deacetylating NAT10 and thereby modulating *ac4C* modification of *Gababr1* mRNA. We identify the SIRT1‐NAT10‐GABABR1 axis as a potential therapeutic target for anesthesia‐related neurotoxicity. By integrating single‐cell transcriptomics, RNA modification profiling, and electrophysiological assessments, our work provides novel insights into the epitranscriptomic regulation underlying POCD. This research not only advances the current understanding of SIRT1 function but also offers promising avenues for developing RNA‐targeted interventions to enhance perioperative cognitive outcomes in the elderly.

## 2 Materials and Methods

All animal experiments were conducted in accordance with the guidelines approved by the Institutional Animal Care and Use Committee (IACUC). Eighteen‐month‐old specific‐pathogen‐free (SPF) male Sprague–Dawley (SD) rats (550–600 g, strain code: 101; Beijing Vital River Laboratory Animal Technology Co. Ltd., China) were housed under controlled conditions (temperature: 22°C ± 2°C, humidity: 50%–60%, 12‐h light/dark cycle) with ad libitum access to standard chow and water. For primary neuronal cultures, hippocampal tissues were harvested from embryonic day 18 (E18) SPF SD rat embryos (Beijing Vital River Laboratory Animal Technology Co. Ltd.). All surgical procedures, including stereotactic viral injections, were performed under anesthesia, and efforts were made to minimize animal suffering.

Detailed procedures, including reagent preparation and experimental protocols, are provided in Supporting Information.

## 3 Results

### 3.1 Elevated NAT10 Expression and mRNA Acetylation in the Hippocampus of Aged Rats With POCD

**FIGURE 1:**
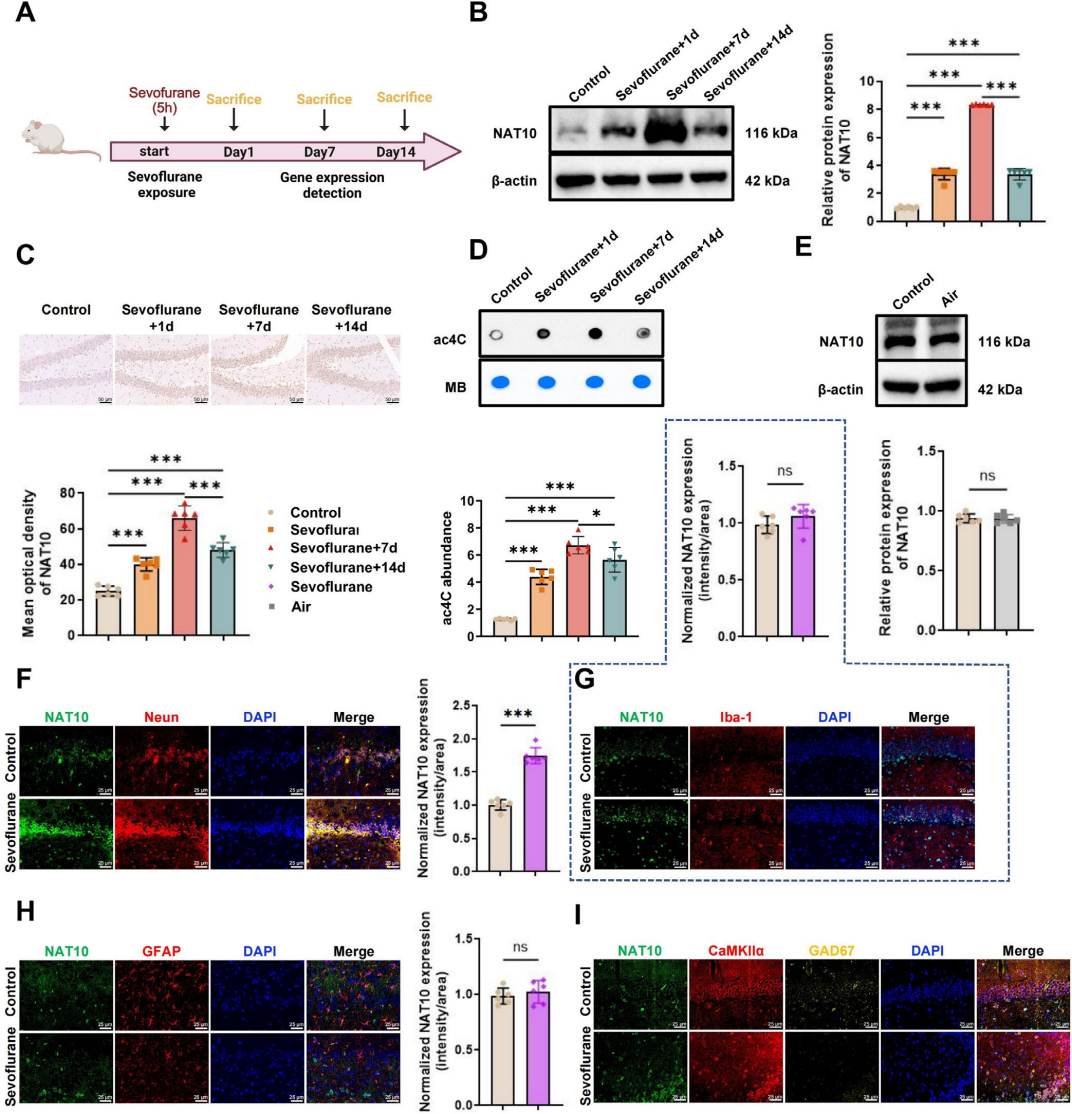
Sevoflurane exposure increases NAT10 expression and mRNA acetylation in the hippocampus of aged rats. (A) Schematic diagram illustrating the experimental workflow for establishing the Sevoflurane‐induced POCD model and subsequent analyses; (B) WB analysis of NAT10 protein expression in hippocampal tissue; (C) immunohistochemical staining of Nat10 in the hippocampal region; scale bar: 50 μm; (D) detection of mRNA acetylation (*ac* ^*4*^ *C*) levels in hippocampal tissue using an *ac* ^*4*^ *C*‐specific antibody; (E) WB comparison of NAT10 protein levels between the air‐exposed group and the control group; (F) immunofluorescent co‐staining showing the localization of NAT10 in NEUN‐positive neurons; scale bar: 25 μm; (G) immunofluorescent co‐staining of NAT10 in Iba‐1‐positive microglia within the DG; scale bar: 25 μm; (H) Immunofluorescent co‐staining of NAT10 in GFAP‐positive astrocytes in the DG; scale bar: 25 μm; (I) immunofluorescent staining of NAT10 expression in CaMKIIα‐positive excitatory neurons and GAD67‐positive inhibitory neurons in the DG. Each group included six animals. ns indicates no statistically significant difference; *p < 0.05, ***p < 0.001.

A sevoflurane‐induced model of POCD was established using 18‐month‐old male rats, randomly assigned to a control group or a sevoflurane exposure group (Figure 1A). WB analysis revealed a significant upregulation of NAT10 protein expression in the hippocampus on days 1 and 7 following sevoflurane exposure, with a partial decline observed by day 14, although levels remained elevated compared to controls (Figure 1B). Immunohistochemical staining corroborated these findings, demonstrating marked increases in NAT10 expression in the hippocampal region on days 1 and 7, with persistent but reduced elevation on day 14 (Figure 1C). To assess whether *Nat10* is involved in mRNA acetylation regulation, hippocampal samples were analyzed using an *ac4C*‐specific antibody. Compared to controls, mRNA *ac4C* modification levels were significantly elevated on days 1 and 7 post‐exposure and declined by day 14, though still higher than baseline (Figure 1D). To exclude the possibility that the observed effects were due to the anesthesia procedure itself, an air‐exposed control group was included. No significant difference in *Nat10* expression was observed between this group and the control, indicating that the changes were specific to sevoflurane exposure (Figure 1E).

To determine the cellular localization of NAT10, double immunofluorescence staining was performed. Results showed increased Nat10 expression in the DG region of Sevoflurane‐exposed rats, predominantly in NEUN‐positive neurons (Figure 1F). No appreciable changes in NAT10 expression were observed in IBA‐1–positive microglia or GFAP‐positive astrocytes (Figure 1G,H). Further analysis using CaMKIIα and GAD67 as markers of excitatory and inhibitory neurons, respectively, revealed that NAT10 expression was primarily localized to CaMKIIα‐positive excitatory neurons, with minimal expression in GAD67‐positive inhibitory neurons (Figure 1I).

Collectively, these findings suggest that sevoflurane exposure induces a robust upregulation of NAT10, specifically in excitatory neurons within the DG region of the hippocampus, accompanied by elevated mRNA *ac4C* modification. This process may contribute to the molecular mechanisms underlying POCD in aged rats.

### 3.2 Neuron‐Specific Knockdown of *Nat10* Alleviates Sevoflurane‐Induced POCD in Aged Rats

To investigate the role of *Nat10* in POCD, AAV‐mediated gene silencing was employed to selectively knock down *Nat10* expression in hippocampal DG neurons of 18‐month‐old rats (Figure S1A). Rats received bilateral injections of rAAV‐hSyn‐EGFP‐sh‐*Nat10* or the control vector rAAV‐hSyn‐*EGFP*‐sh‐NC into the DG region, and transgene expression was confirmed 3 weeks post‐injection. RT‐qPCR analysis demonstrated a significant reduction in *Nat10* mRNA levels in the sevoflurane + sh‐Nat10 group compared to the sevoflurane + sh‐NC group (Figure S1B).

WB and immunofluorescence assays revealed that NAT10 protein levels were markedly elevated in the sevoflurane and sevoflurane + sh‐NC groups relative to the control group, whereas NAT10 expression was significantly suppressed in the sevoflurane + sh‐Nat10 group (Figure S1C–E). Immunofluorescence for EGFP confirmed accurate viral targeting and infection localized predominantly within DG neurons (Figure S1F).

Behavioral assessments were conducted 12–14 days following sevoflurane exposure. Tests included the OFT, Y‐maze, NORT, and MWM. OFT results indicated no significant differences in locomotor activity among groups (Figure S2A,B). In contrast, Y‐maze analysis showed that the sevoflurane and sevoflurane + sh‐NC groups exhibited significantly reduced alternation percentages compared to the control group, an effect reversed by Nat10 knockdown (Figure S2C). Similarly, NORT revealed that the sevoflurane and sevoflurane + sh‐NC groups had decreased novel object exploration time and lower discrimination indices, both of which were significantly restored in the sevoflurane + sh‐Nat10 group (Figure S2D). MWM performance further confirmed cognitive deficits in the sevoflurane and sevoflurane + sh‐NC groups, as evidenced by prolonged escape latency during training, which was significantly shortened in the sevoflurane + sh‐Nat10 group (Figure S2E,F).

Collectively, these findings suggest that targeted knockdown of Nat10 in DG neurons effectively mitigates sevoflurane‐induced impairments in learning and memory in aged rats.

### 3.3 Silencing *Nat10* Enhances Mitochondrial Autophagy and Alleviates Cellular Energy Stress in Neurons

**FIGURE 2:**
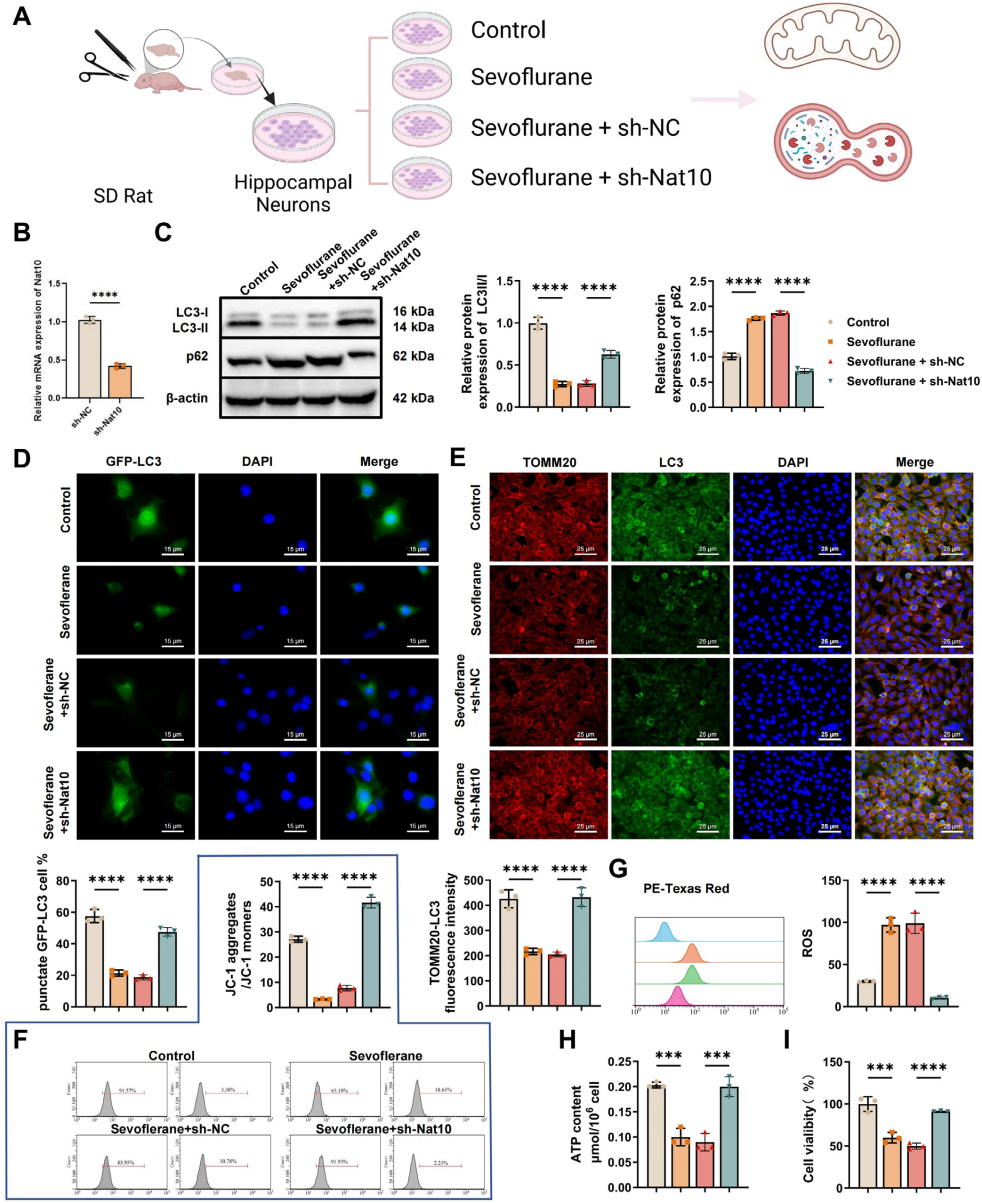
Effects of *Nat10* knockdown on neuronal mitophagy and energy metabolism. (A) Schematic overview of the experimental workflow for Nat10 knockdown in primary neurons and subsequent assessment of mitophagy and cellular homeostasis; (B) RT‐qPCR was performed to examine the expression level of *Nat10* in neurons; (C) WB analysis of LC3‐II/LC3‐I ratio and P62 protein expression in neurons; (D) immunofluorescence imaging of GFP‐LC3 puncta formation (scale bar: 15 μm); (E) co‐localization analysis of LC3 and TOM20 by immunofluorescence (scale bar: 25 μm); (F) JC‐1 staining to assess changes in MMP; (G) flow cytometry‐based quantification of intracellular ROS levels; (H) colorimetric assay to determine intracellular ATP content; (I) CCK‐8 assay to evaluate neuronal viability. All cell‐based experiments were performed in triplicate. ***p < 0.001, ****p < 0.0001, compared between indicated groups.

Primary hippocampal neurons were used to investigate the effects of *Nat10* knockdown on mitochondrial autophagy and energy metabolism (Figure 2A). The knockout efficiency of sh‐Nat10 in hippocampal neurons was assessed using RT‐qPCR. The results showed that sh‐Nat10 effectively reduced the expression of the *Nat10* gene in hippocampal neurons (Figure 2B). WB analysis revealed that, compared with the Control group, both the sevoflurane and sevoflurane + sh‐NC groups exhibited a significantly reduced LC3‐II/LC3‐I ratio and increased P62 expression. In contrast, Nat10 silencing (sevoflurane + sh‐Nat10) markedly elevated the LC3‐II/LC3‐I ratio and decreased P62 levels (Figure 2C). Immunofluorescence analysis following GFP‐LC3 transfection showed a notable increase in GFP‐LC3 puncta formation in the Nat10‐silenced group relative to the Control, whereas both sevoflurane‐treated groups without *Nat10* knockdown exhibited a pronounced reduction (Figure 2D). Additionally, co‐localization of LC3 with the mitochondrial marker TOMM20 was significantly enhanced in the sevoflurane + sh‐Nat10 group, while diminished signals were observed in the other sevoflurane‐exposed groups (Figure 2E), suggesting that *Nat10* knockdown promotes mitochondrial autophagy.

To further assess mitochondrial function, JC‐1 staining was employed to evaluate the MMP. Results demonstrated a substantial decline in the red/green fluorescence ratio in the sevoflurane and sevoflurane + sh‐NC groups, whereas *Nat10* knockdown effectively restored this parameter (Figure 2F). Flow cytometric analysis of intracellular ROS revealed elevated ROS levels following Sevoflurane exposure, which was significantly reduced upon *Nat10* silencing (Figure 2G). Similarly, ATP quantification using a colorimetric assay showed that ATP content decreased in the sevoflurane and sevoflurane + sh‐NC groups but was significantly elevated in the sevoflurane + sh‐Nat10 group (Figure 2H). Furthermore, cell viability assessed by CCK‐8 following 2‐DG treatment indicated a notable decline in metabolic activity in the sevoflurane‐exposed neurons, which was significantly improved by *Nat10* knockdown (Figure 2I).

Collectively, these findings indicate that *Nat10* knockdown enhances mitochondrial autophagy, mitigates cellular energy stress, and contributes to the maintenance of neuronal homeostasis and survival under sevoflurane‐induced stress conditions.

### 3.4 Identification of DEGs in Hippocampal Neurons With High and Low Autophagy Scores via Single‐Cell Sequencing

**FIGURE 3:**
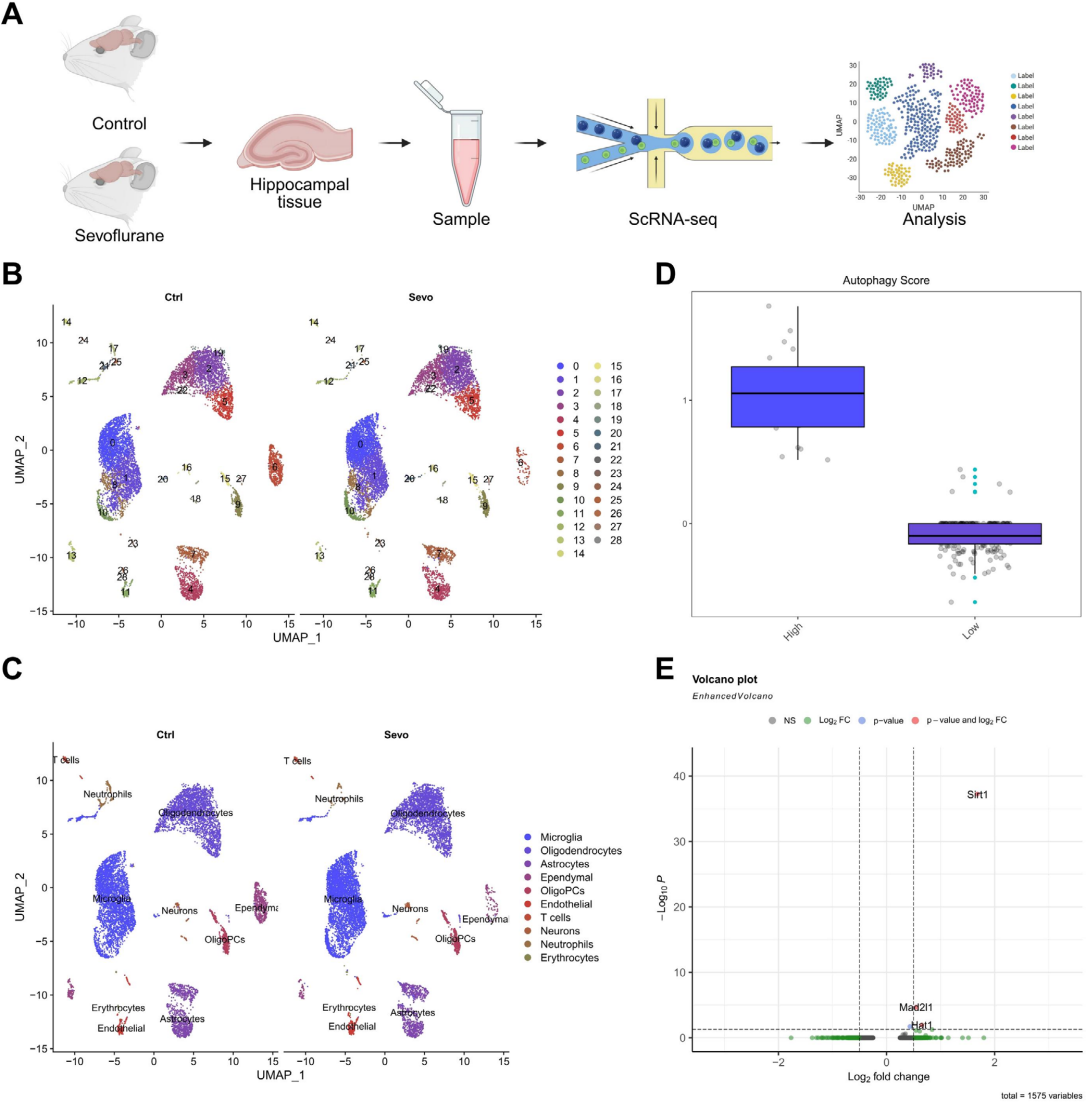
scRNA‐seq reveals sevoflurane‐induced alterations in hippocampal cell heterogeneity and autophagy‐related gene expression in rats. (A) Experimental workflow for scRNA‐seq of hippocampal tissue from control and Sevoflurane‐treated rats, including tissue dissociation, library preparation, sequencing, and data analysis; (B) UMAP dimensionality reduction showing cell cluster distributions in the Ctrl and Sevo groups; (C) UMAP projection indicating major annotated cell types, including microglia, oligodendrocytes, astrocytes, and neurons; (D) box plot depicting the classification of neurons into high and low autophagy subpopulations based on autophagy scoring; (E) Volcano plot illustrating DEGs between high‐ and low‐autophagy neuronal subgroups. Each group included three animals.

Single‐cell suspensions were successfully isolated from the hippocampal tissues of control and sevoflurane‐treated rats, followed by high‐throughput scRNA‐seq to profile the transcriptomic landscape (Figure 3A). Quality control analysis revealed comparable distributions between groups in terms of cell cycle phase scores, mitochondrial gene content, and transcript counts, indicating robust data quality (Figure S3A–C). Dimensionality reduction using highly variable gene selection and Harmony integration demonstrated distinct transcriptomic separation between the two groups, suggesting that sevoflurane exposure may induce transcriptional reprogramming in hippocampal cells (Figure S3D,E).

Subsequent data integration and clustering with the Seurat package, visualized via UMAP, identified 29 distinct cellular clusters distributed across both groups (Figure 3B). These clusters were classified into 10 major cell types based on canonical marker expression: neurons (Kif5c, Camk2a, Snap25, Cplx2, Gap43), microglia (C1qc, Ly86, C1qa, C1qb, Ctss), astrocytes (Slc4a4, Cldn10, Gjb6, Btbd17, Agt), oligodendrocyte precursor cells (OligoPCs; Pdgfra, Lhfpl3, C1ql1, Cacng4, Vcan), mature oligodendrocytes (Mog, Ermn, Mal, Opalin, Plp1), endothelial cells (Cldn5, Ly6c1, Flt1, Ptprb, Esam), ependymal cells (Calml4, Folr1, Dynlrb2, Lbp, Pifo), T cells (Cd2, Cd3g, Cd3d, Cd3e, Gimap3), neutrophils (S100a9, S100a8, Mmp9, Mmp8, Slpi), and erythrocytes (Hba‐a2, Hba‐a1, Hbb‐bs, Hbb‐bt, Alas2) (Figure 3C; Figure S3F,G). To assess autophagic activity, a module score‐based model was constructed using the AddModuleScore function, enabling the stratification of neurons into high‐autophagy and low‐autophagy subpopulations. A significant difference in autophagy activity was observed between the two groups (Figure 3D). Differential gene expression analysis further revealed a marked upregulation of *Sirt1* in neurons with high autophagy scores, implying a potential role for sevoflurane in promoting neuronal homeostasis via *Sirt1*‐mediated autophagic pathways (Figure 3E). This finding is consistent with previous studies demonstrating the neuroprotective functions of *Sirt1* through autophagy modulation in neurological disorders [12].

### 3.5 SIRT1 Directly Binds and Deacetylates NAT10

**FIGURE 4:**
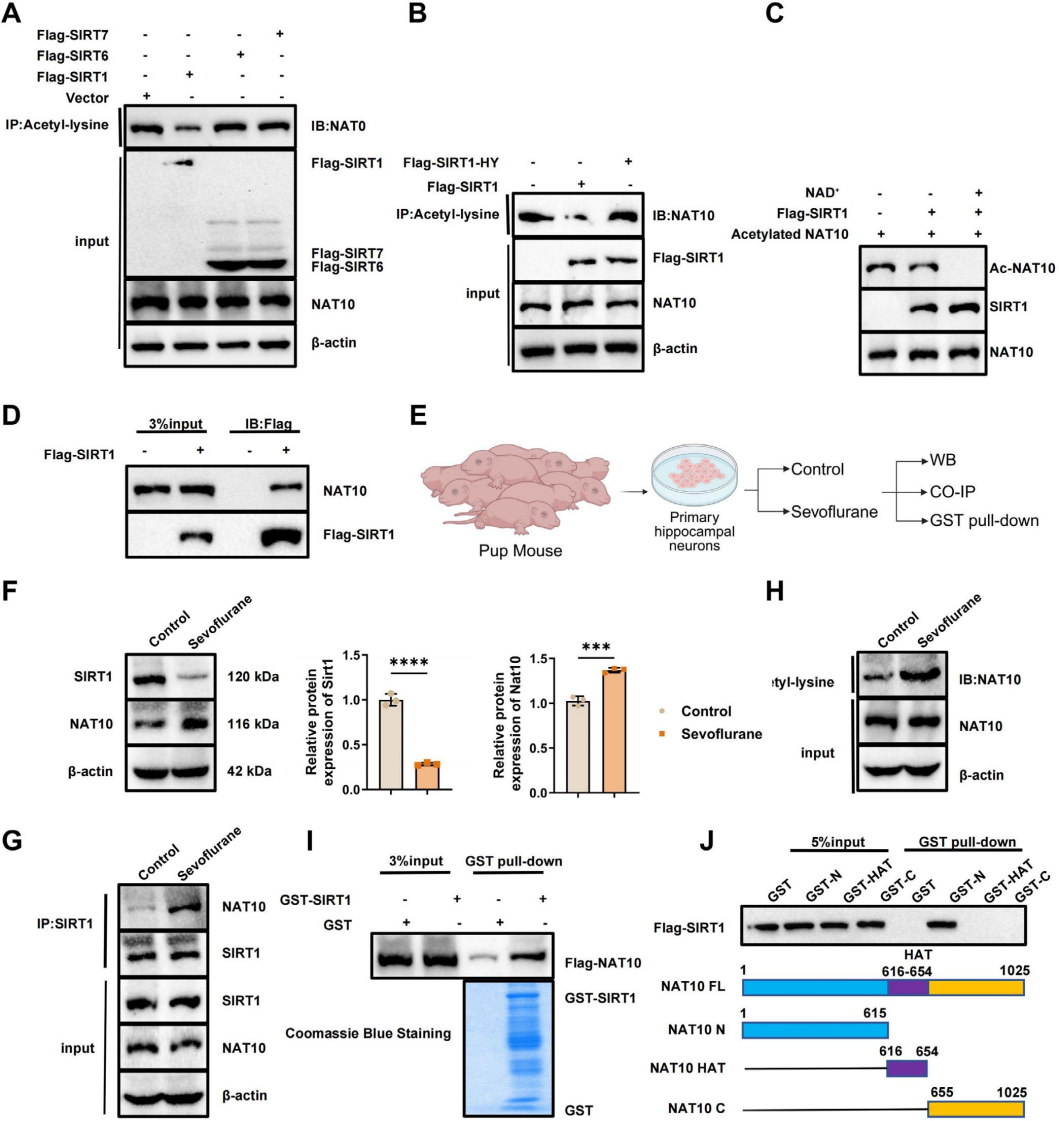
Deacetylation of NAT10 by SIRT1. (A) WB analysis of NAT10 acetylation levels following overexpression of SIRT1, SIRT6, and SIRT7; (B) effect of the enzymatically inactive SIRT1 mutant (H363Y) on NAT10 acetylation, assessed by WB; (C) in vitro deacetylation assay showing the regulatory effect of purified Flag‐SIRT1 on His‐NAT10 acetylation; (D) Co‐IP demonstrating the interaction between SIRT1 and NAT10 in cells; (E) schematic overview of the experimental design used to evaluate the SIRT1‐NAT10 interaction and NAT10 acetylation under sevoflurane exposure; (F) WB analysis of SIRT1 and NAT10 expression in hippocampal neurons from control and sevoflurane‐exposed groups; (G) co‐IP confirming the enhanced interaction between SIRT1 and NAT10 following sevoflurane treatment; (H) Immunoprecipitation detecting changes in NAT10 acetylation levels; (I) GST pull‐down assay confirming the direct interaction between GST‐SIRT1 and Flag‐NAT10; (J) mapping of the binding region between SIRT1 and distinct NAT10 domains using GST pull‐down assays. All experiments were performed in triplicate. ***p < 0.001; ****p < 0.0001.

Previous studies have suggested that among members of the Sirtuin family, SIRT1 uniquely regulates the acetylation status of NAT10. To verify this specificity, SIRT1, SIRT6, and SIRT7 were individually overexpressed, and changes in NAT10 acetylation levels were assessed. WB analysis revealed that only SIRT1 significantly reduced NAT10 acetylation, whereas SIRT6 and SIRT7 had no observable effect (Figure 4A). Furthermore, a catalytically inactive mutant of SIRT1 (H363Y) failed to reduce NAT10 acetylation levels, underscoring the requirement of its deacetylase activity (Figure 4B). In vitro deacetylation assays using purified Flag‐tagged SIRT1 and pre‐acetylated His‐tagged NAT10 demonstrated that SIRT1 effectively deacetylates NAT10 in an NAD^+^‐dependent manner (Figure 4C). Co‐IP assays further confirmed a physical interaction between SIRT1 and NAT10 in cellular contexts (Figure 4D).

Building on these findings, we next examined the interaction and regulatory dynamics between *Sirt1* and *Nat10* under sevoflurane exposure (Figure 4E). Primary hippocampal neurons were divided into control and sevoflurane‐treated groups. WB revealed a marked decrease in SIRT1 expression and a concomitant increase in NAT10 levels in the sevoflurane group compared to controls (Figure 4F). Co‐IP analysis confirmed the interaction between SIRT1 and NAT10 in both groups, with a stronger binding observed following sevoflurane treatment (Figure 4G).

To assess the functional consequence of this interaction, immunoprecipitation assays were conducted to measure NAT10 acetylation. sevoflurane exposure led to a significant increase in NAT10 acetylation compared to control (Figure 4H). Additionally, GST pull‐down assays demonstrated a direct physical interaction between GST‐tagged SIRT1 and purified Flag‐NAT10, whereas GST alone failed to bind NAT10 (Figure 4I). Truncation mutants of NAT10 revealed that SIRT1 predominantly interacts with its N‐terminal domain but not with the HAT or C‐terminal regions (Figure 4J).

Collectively, these results demonstrate that SIRT1 directly binds to and deacetylates NAT10, and that this interaction is modulated by sevoflurane exposure. This suggests a mechanistic role for SIRT1‐mediated deacetylation of NAT10 in the context of anesthesia‐induced neurotoxicity.

### 3.6 SIRT1 Enhances Autophagy and Mitochondrial Metabolism to Preserve Neuronal Homeostasis by Suppressing *Nat10* Expression

**FIGURE 5:**
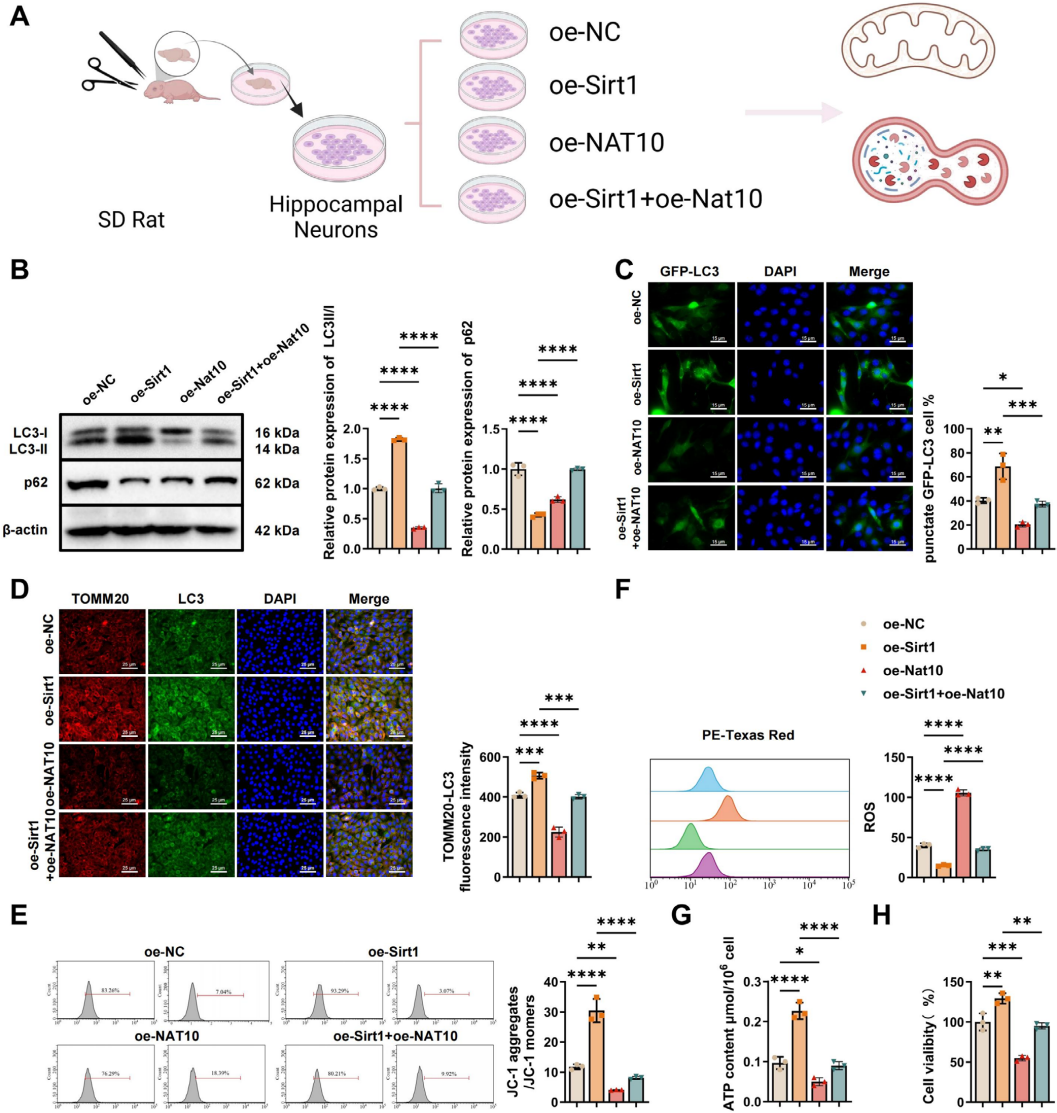
Effects of *SIRT1/NAT10* on autophagy, energy metabolism, and neuronal homeostasis. (A) Schematic diagram outlining the experimental design for assessing autophagy and cellular homeostasis following *Sirt1* and *Nat10* overexpression; (B) WB analysis of autophagy‐related proteins LC3‐II/LC3‐I and P62 in neurons; (C) immunofluorescence quantification of GFP‐LC3 puncta‐positive cells, bar = 15 μm; (D) immunofluorescence imaging of LC3 and TOMM20 colocalization, bar = 25 μm; (E) JC‐1 staining to assess MMP by red/green fluorescence ratio; (F) flow cytometry analysis of intracellular ROS levels; (G) colorimetric assay of cellular ATP content; (H) CCK‐8 assay of cell viability following 2‐DG treatment. All cell experiments were performed in triplicate. *p < 0.05, **p < 0.01, ***p < 0.001 indicate statistical significance between groups.

To investigate the interplay between SIRT1 and *Nat10* in regulating neuronal autophagy and metabolic stability under sevoflurane exposure, primary hippocampal neurons were transfected to overexpress either *Sirt1*, *Nat10*, or both. Cells were divided into four groups: oe‐NC (negative control), oe‐Sirt1, oe‐Nat10, and oe‐Sirt1 + oe‐Nat10, with all groups subjected to sevoflurane treatment (Figure 5A).

WB analysis revealed that overexpression of SIRT1 significantly increased the LC3‐II/LC3‐I ratio and decreased P62 levels, indicating enhanced autophagic activity. In contrast, NAT10 overexpression produced the opposite effect, reducing the LC3‐II/LC3‐I ratio and elevating P62 expression. Notably, co‐overexpression of SIRT1 and NAT10 attenuated the autophagy‐promoting effect of SIRT1 alone (Figure 5B). Immunofluorescence staining of GFP‐LC3 further supported these findings: SIRT1 increased the proportion of cells with punctate LC3 aggregation, whereas NAT10 reduced it; the combination of both resulted in diminished LC3 puncta compared to Sirt1 overexpression alone (Figure 5C). To assess mitophagy, LC3 and TOMM20 co‐localization was examined. SIRT1 overexpression markedly enhanced their overlap, while NAT10 reduced the signal intensity. This enhancement was again diminished when both genes were co‐expressed, suggesting that NAT10 counteracts the mitophagy‐promoting effects of SIRT1 (Figure 5D).

Further evaluation of mitochondrial function showed that SIRT1 increased MMP, as measured by JC‐1 staining, whereas NAT10 decreased it. Co‐expression of both resulted in a significant reduction in MMP relative to SIRT1 alone (Figure 5E). Similarly, flow cytometry demonstrated that SIRT1 reduced intracellular ROS levels while NAT10 increased them. In the co‐overexpression group, ROS levels were significantly higher than in the SIRT1‐only group (Figure 5F). ATP content, measured by a colorimetric assay, was elevated in the SIRT1 group and suppressed by NAT10, with co‐expression again reversing the beneficial effects of SIRT1 (Figure 5G). CCK‐8 assays performed after 2‐DG treatment revealed that SIRT1 enhanced neuronal viability, NAT10 impaired it, and dual overexpression mitigated the protective effect of SIRT1 (Figure 5H).

Collectively, these results demonstrate that SIRT1 promotes neuronal mitophagy, improves mitochondrial function, alleviates metabolic stress, and supports cell survival under anesthetic exposure. Conversely, NAT10 overexpression antagonizes these protective effects, suggesting a regulatory balance between the two proteins in maintaining neuronal homeostasis.

### 3.7 *Nat10* Enhances *Gababr1* Expression via mRNA Acetylation

**FIGURE 6:**
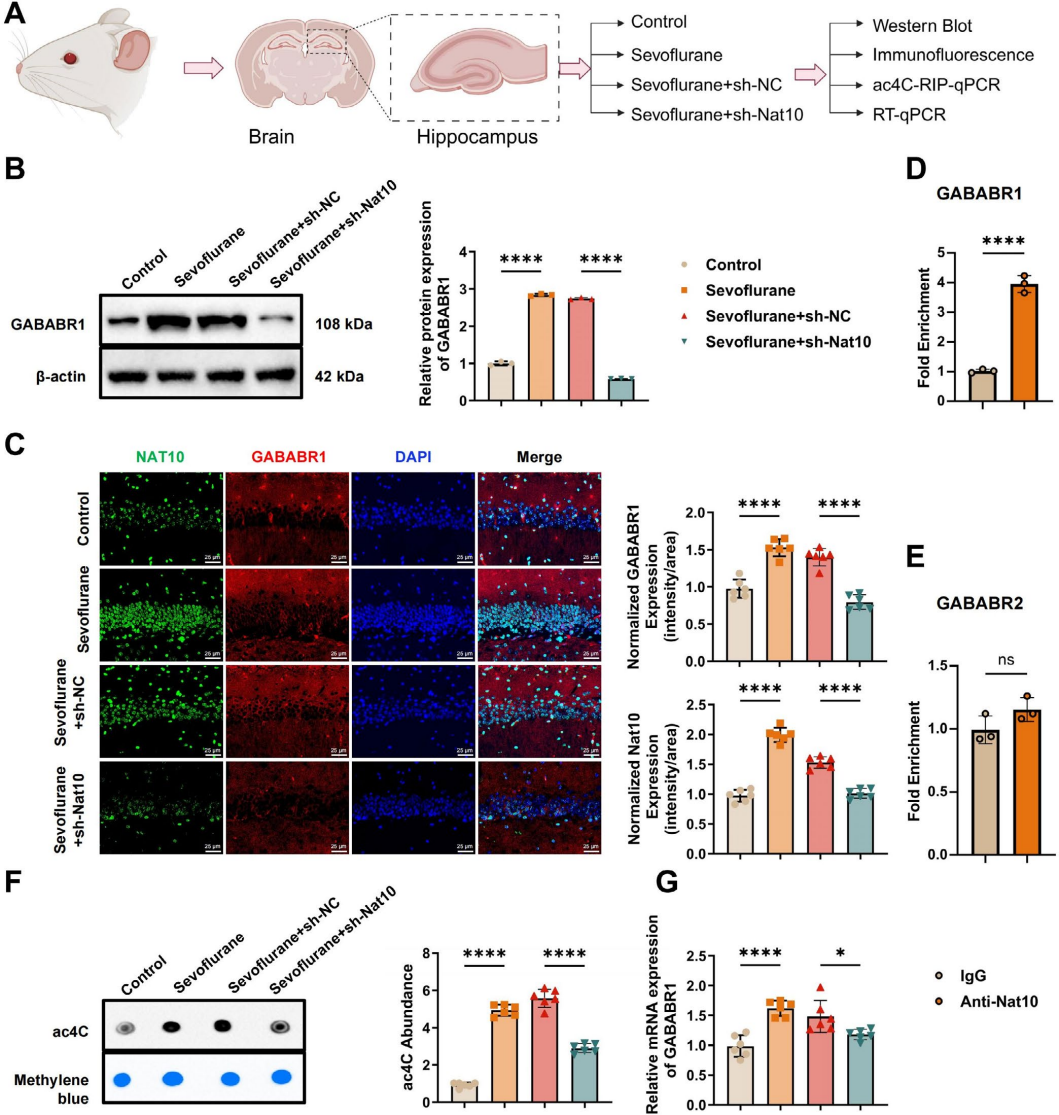
*Nat10* mediates GABABR1 expression via mRNA acetylation. (A) Schematic overview of the experimental design investigating NAT10‐mediated regulation of GABABR1 in the Sevoflurane exposure model; (B) WB analysis of GABABR1 protein expression in hippocampal tissue; (C) immunofluorescence staining showing the localization and expression of GABABR1 in the hippocampal DG region (scale bar: 25 μm); (D) RIP‐qPCR assessing the binding of NAT10 to *Gababr1* mRNA; (E) RIP‐qPCR evaluating the interaction between NAT10 and *Gababr2* mRNA; (F) Dot blot analysis of global mRNA acetylation levels in hippocampal tissue; (G) RT‐qPCR quantification of *Gababr1* mRNA expression. Each group consisted of six animals; in vitro experiments were independently repeated three times. *p < 0.05, ****p < 0.0001; ns indicates no statistically significant difference.

Previous research [13] has indicated that *Nat10* regulates *Gababr1* expression through mRNA acetylation. To further investigate this mechanism in the context of sevoflurane‐induced cognitive impairment, we systematically examined the role of *Nat10* in modulating *Gababr1* expression using hippocampal tissues and primary neurons from four experimental groups: control, sevoflurane, sevoflurane + sh‐NC, and sevoflurane + sh‐Nat10 (Figure 6A).

WB analysis revealed a significant upregulation of GABABR1 protein levels in the sevoflurane and sevoflurane + sh‐NC groups compared to controls, while knockdown of Nat10 (sevoflurane + sh‐Nat10) markedly reduced its expression (Figure 6B). Immunofluorescence staining further confirmed these findings and demonstrated that GABABR1 is predominantly localized within neuronal soma in the DG region (Figure 6C). To assess the molecular interaction between *Nat10* and *Gababr1/2* transcripts, we performed RIP‐qPCR. The results showed significant enrichment of *Gababr1* mRNA in *Nat10* immunoprecipitates, indicating a specific binding interaction (Figure 6D). In contrast, no significant association was detected between *Nat10* and *Gababr2* mRNA (Figure 6E), suggesting selective regulation.

Dot blot analysis of hippocampal mRNA acetylation levels revealed that sevoflurane exposure significantly increased overall ac4C modification, an effect reversed by Nat10 knockdown (Figure 6F). Consistently, RT‐qPCR results showed that *Gababr1* mRNA expression was upregulated by sevoflurane and normalized by *Nat10* suppression (Figure 6G).

Collectively, these findings demonstrate that *Nat10* directly binds to *Gababr1* mRNA and promotes its acetylation, thereby enhancing both transcript and protein expression. This regulatory mechanism likely contributes to the molecular pathology of sevoflurane‐induced cognitive dysfunction in hippocampal neurons.

### 3.8 SIRT1 Overexpression Attenuates GABABR1‐Mediated Inhibitory Synaptic Currents via Nat10 Suppression

Eighteen‐month‐old male Sprague–Dawley rats were randomly assigned to four groups: oe‐NC, oe‐Sirt1, oe‐Nat10, and oe‐Sirt1 + oe‐Nat10. Each group received a stereotaxic injection into the hippocampal DG with either AAV‐hSyn‐EGFP‐oe‐Sirt1, AAV‐hSyn‐EGFP‐oe‐Nat10, AAV‐hSyn‐EGFP‐oe‐Sirt1 + oe‐Nat10, or a control virus (AAV‐hSyn‐EGFP‐oe‐NC), followed by exposure to sevoflurane (Figure S4A).

Immunofluorescence imaging confirmed that EGFP expression was predominantly localized to neurons within the DG region (Figure S4B). WB analysis revealed that SIRT1 protein levels were significantly increased, whereas NAT10 expression was suppressed in the oe‐Sirt1 group relative to oe‐NC. In contrast, NAT10 expression was markedly upregulated in the oe‐Nat10 group, with no observable change in SIRT1 levels. Notably, in the oe‐Sirt1 + oe‐Nat10 group, NAT10 expression was restored despite continued SIRT1 overexpression, indicating a regulatory interplay between the two proteins (Figure S4C).

Further assessment of GABABR1 expression in the DG via WB and RT‐qPCR showed that SIRT1 overexpression significantly reduced GABABR1 levels, while NAT10 overexpression enhanced them. Co‐expression of SIRT1 and NAT10 partially reversed the Sirt1‐induced downregulation of GABABR1 (Figure S4C,D). Immunofluorescence confirmed that GABABR1 was primarily localized to the somata of DG neurons and that expression patterns were consistent with WB findings (Figure S4E).

To evaluate the functional consequences of these molecular changes, whole‐cell patch‐clamp recordings were conducted on DG granule cells to measure IPSCs. Application of 300 μM Baclofen elicited inward currents (IBaclofen), which were significantly reduced in the oe‐Sirt1 group but enhanced in the oe‐Nat10 group, compared to controls. Notably, co‐expression of SIRT1 and NAT10 restored IBaclofen amplitudes (Figure S5A). The application of CGP52432 (30 μM), a selective GABABR antagonist, abolished these currents, confirming GABABR‐mediated origin (Figure S5A). Additionally, IPSC_slow was induced by 10‐pulse 66 Hz stimulation under pharmacological isolation (picrotoxin, NBQX, and D‐APV). The oe‐Sirt1 group exhibited significantly attenuated IPSC_slow amplitudes, whereas the oe‐Nat10 group showed increased responses. Co‐expression of SIRT1 and NAT10 reversed the Sirt1‐mediated suppression (Figure S5B). CGP52432 completely blocked IPSC_slow, reaffirming GABABR specificity (Figure S5B).

Collectively, these findings indicate that SIRT1 mitigates sevoflurane‐induced inhibitory synaptic transmission by downregulating NAT10, thereby reducing GABABR1 expression and GABABR‐mediated IPSCs. Importantly, NAT10 overexpression counteracts this effect, highlighting the critical role of the SIRT1‐NAT10‐GABABR1 axis in modulating inhibitory synaptic signaling.

### 3.9 SIRT1 Overexpression Enhances Autophagy and Restores Cognitive Function by Suppressing *Nat10* and Improving Energy Metabolism

Eighteen‐month‐old male SD rats were randomly assigned to four groups: oe‐NC (control), oe‐Sirt1, oe‐Nat10, and oe‐Sirt1 + oe‐Nat10. Recombinant AAVs—AAV‐hSyn‐EGFP‐oe‐Sirt1, AAV‐hSyn‐EGFP‐oe‐Nat10, AAV‐hSyn‐EGFP‐oe‐Sirt1 + oe‐Nat10, and the control vector AAV‐hSyn‐EGFP‐oe‐NC—were stereotactically injected into the hippocampal DG. Three weeks post‐injection, after confirmed viral expression, all groups were subjected to sevoflurane exposure (Figure S6A).

To assess the impact of SIRT1/*Nat10* modulation on hippocampal autophagy and cellular homeostasis, WB analysis was performed to quantify LC3‐II/LC3‐I ratios and P62 protein levels in DG tissue. Compared to the oe‐NC group, oe‐Sirt1 rats exhibited significantly increased LC3‐II/LC3‐I ratios and decreased P62 expression, indicative of enhanced autophagic activity. In contrast, oe‐Nat10 rats showed the opposite trend. Co‐overexpression of *Sirt1* and *Nat10* (oe‐Sirt1 + oe‐Nat10) partially reversed the effects of SIRT1 alone, with decreased LC3‐II/LC3‐I ratios and elevated P62 levels (Figure S6B). Energy metabolism was evaluated by measuring ATP concentrations using a luciferin–luciferase assay. ATP levels were significantly elevated in the oe‐Sirt1 group compared to oe‐NC, but markedly reduced in the oe‐Nat10 group. Notably, ATP content in the oe‐Sirt1 + oe‐Nat10 group was also significantly lower than in the oe‐Sirt1 group alone (Figure S6C). Similarly, JC‐1 staining revealed that MMP, reflected by the red/green fluorescence ratio, increased in oe‐Sirt1 rats and decreased in the oe‐Nat10 group. Co‐overexpression again led to reduced MMP compared to oe‐Sirt1 alone (Figure S6D).

Cognitive performance was assessed via Y‐maze, NORT, and the MWM. In the Y‐maze, spontaneous alternation rates were significantly higher in oe‐Sirt1 rats and lower in oe‐Nat10 rats relative to controls; the oe‐Sirt1 + oe‐Nat10 group showed significantly reduced alternation rates compared to the oe‐Sirt1 group (Figure S6E,F). In the NORT, discrimination indices followed a similar pattern, with oe‐Sirt1 rats demonstrating improved object recognition, which was diminished by *Nat10* co‐overexpression (Figure S6G). The MWM revealed that oe‐Sirt1 rats exhibited shorter escape latencies and more platform crossings, suggesting enhanced spatial learning and memory. These effects were reversed by *Nat10* overexpression, which increased escape latency and decreased platform crossings (Figure S6H,I).

In summary, overexpression of SIRT1 enhances autophagy, improves mitochondrial function, and restores cognitive performance in aged rats following sevoflurane exposure, primarily by suppressing *Nat10* expression. Conversely, *Nat10* overexpression negates these protective effects, underscoring the critical regulatory role of the SIRT1‐*Nat10* axis in POCD.

## 4 Discussion

This study reveals that SIRT1 alleviates sevoflurane‐induced POCD by deacetylating NAT10, thereby reducing ac4C modification of *Gababr1* mRNA and restoring synaptic/autophagy balance. While SIRT1's roles in oxidative stress, autophagy, and neuroprotection are well‐documented [14, 15, 16, 17], this work uncovers its novel function in regulating RNA‐modifying enzymes. The findings establish a direct SIRT1‐NAT10‐GABABR1 axis linking mRNA acetylation to synaptic dysfunction, expanding SIRT1's therapeutic potential for anesthesia‐related neurotoxicity.

*Nat10*, the sole known mRNA *ac4C* writer, has established roles in cancer and senescence [18, 19] but emerging nervous system functions [8, 20]. This study reveals sevoflurane upregulates hippocampal *Nat10* and *ac4C* levels, enhancing *Gababr1* expression through direct mRNA binding/acetylation—establishing its novel role in anesthesia‐induced synaptic dysfunction. These findings significantly expand *ac4C* epitranscriptomics' relevance in CNS pathology.

GABABR1 is essential for inhibitory synaptic transmission [21, 22] and its dysregulation contributes to CNS disorders including cognitive deficits [23, 24]. While GABAergic dysfunction in POCD is well‐documented, this study reveals *Nat10*‐mediated *ac4C* modification of *Gababr1* mRNA as a novel upstream regulatory mechanism that enhances Baclofen‐induced IPSCs. Conversely, SIRT1 counteracts this effect by reducing *ac4C* modification. These findings establish the first direct link between mRNA acetylation and inhibitory synaptic dysfunction in sevoflurane‐induced POCD.

This study demonstrates that SIRT1 improves mitochondrial quality control in POCD by enhancing autophagy (increased LC3‐II and LC3/TOM20 co‐localization) and restoring energy homeostasis (reduced ROS, elevated ATP). These neuroprotective effects are mediated through SIRT1's deacetylation of *Nat10*, as *Nat10* overexpression reverses the benefits. The findings reveal an unexpected role for RNA modifications (via the SIRT1‐NAT10 axis) in regulating organelle function, establishing a novel multi‐level regulatory mechanism with therapeutic potential for POCD.

This study further demonstrates that SIRT1 improves mitochondrial quality control in POCD by promoting autophagy, as evidenced by increased LC3‐II levels and enhanced LC3/TOM20 co‐localization. In addition, SIRT1 restores energy homeostasis by reducing reactive oxygen species (ROS) levels and enhancing ATP production. These neuroprotective effects depend on the deacetylation of NAT10 by SIRT1, as overexpression of NAT10 reverses these benefits. To further verify the role of autophagy in SIRT1‐mediated mitochondrial protection, future studies will inhibit autophagy using pharmacological inhibitors such as chloroquine or 3‐methyladenine (3‐MA) to assess whether autophagy is essential for restoring mitochondrial function—specifically the reduction of ROS and the increase in ATP levels—under conditions of SIRT1 overexpression. Previous studies have shown that SIRT1 regulates autophagy and mitochondrial quality control by deacetylating multiple pathways, including FOXO, ATGs, and LC3, thereby alleviating oxidative stress and improving energy metabolism, suggesting a causal link between the SIRT1‐autophagy axis and mitochondrial homeostasis [25]. In ischemia/hypoxia‐related models, inhibition of SIRT1/2 eliminates the beneficial effects of pro‐autophagic interventions on mitophagy markers and mitochondrial function, further confirming the critical role of SIRT1‐dependent autophagy in mitochondrial protection [26]. Moreover, recent mechanistic studies in endometrial carcinoma have shown that SIRT1 promotes BNIP3 transcription by deacetylating FOXO3, thereby activating PINK1/Parkin‐mediated mitophagy; disruption of this pathway attenuates SIRT1‐associated mitochondrial phenotypes [27]. Therefore, our subsequent research will build upon this direction to further validate the specific role of autophagy in SIRT1‐mediated mitochondrial protection.

This study reveals that SIRT1 regulates the *ac* ^*4*^ *C* modification of *GABABR1* mRNA by deacetylating NAT10, thereby ameliorating sevoflurane‐induced POCD. This finding provides a potential therapeutic strategy targeting the SIRT1‐NAT10 axis. Although this study successfully confirmed that SIRT1 modulates NAT10 function through deacetylation, the specific lysine residue targeted by SIRT1 remains unidentified. Based on previous reports, the most likely candidate site is K426 of NAT10, which has been verified as a self‐acetylation site critical for its function in rRNA transcription and protein substrate acetylation; mutation of this residue (K426R) abolishes these functions [28]. Moreover, during energy stress, SIRT1‐mediated deacetylation drives a cellular shift from rRNA biogenesis toward autophagy [29]. This process coincides with changes in NAT10 acetylation status, and in mechanistic experiments, the “NAT10‐KR” mutant exhibited phenotypes consistent with deacetylation, further supporting the hypothesis that SIRT1 targets NAT10 at or near K426. Although this study elucidates the mechanism by which SIRT1 improves cognitive function via NAT10 deacetylation, direct genetic evidence confirming whether the neuroprotective effect of SIRT1 is entirely dependent on this pathway is still lacking. Future research could employ an “acetylation‐mimic” NAT10 mutant by introducing site‐specific mutations to simulate the acetylated state. In addition, although we demonstrated the interaction between NAT10 and *GABABR1* mRNA, the precise *ac* ^*4*^ *C* modification site has not yet been identified, representing a limitation of this study. Future studies may utilize mass spectrometry or single‐molecule quantitative approaches combined with RNA modification–specific antibodies to pinpoint *ac* ^*4*^ *C* sites on *GABABR1* mRNA. Such findings would provide a more precise molecular mechanism for the role of NAT10 in synaptic regulation and establish a stronger theoretical foundation for its potential applications in POCD and other neurological disorders.

This study focuses on sevoflurane‐induced POCD and elucidates that SIRT1 ameliorates POCD by regulating the *ac* ^*4*^ *C* modification of *GABABR1* mRNA through deacetylation of NAT10. This finding provides a potential therapeutic strategy targeting the SIRT1‐NAT10 axis. For example, other commonly used anesthetics, such as isoflurane and desflurane, may influence cognitive function through similar mechanisms involving SIRT1‐NAT10 regulation. Notably, SIRT1 activators such as resveratrol have been shown to exert neuroprotective, pro‐autophagic, and anti‐aging effects [30] and may contribute to the prevention of POCD by enhancing SIRT1 activity. Meanwhile, the development of agents capable of effectively inhibiting NAT10 function may offer novel therapeutic options for the prevention and treatment of POCD. Furthermore, this signaling axis may also participate in the cognitive impairments observed in neurodegenerative diseases such as Alzheimer's disease and Parkinson's disease [31, 32]. Therefore, future studies should further explore how these established or emerging pharmacological agents can be applied clinically to modulate the SIRT1‐NAT10 axis, thereby improving neural function and preventing or treating POCD (Graphical Abstract).

## Author Contributions

X.X. and W.D.: conceptualization, methodology, investigation, data curation, writing – original draft. Y.Z. and X.Z.: conceptualization, supervision, project administration, funding acquisition, writing – review and editing. All authors reviewed and approved the final manuscript.

## Funding

This study was supported by the Liaoning Provincial Doctoral Research Start‐up Foundation (No. 2023‐BS‐044).

## Ethics Statement

All animal experiments were approved by the Animal Ethics Committee of Cancer Hospital of China Medical University (Approval No. CMUKT2024179).

## Conflicts of Interest

The authors declare no conflicts of interest.

## Supporting information


**Figure S1:** Validation of *Nat10* knockdown in hippocampal DG neurons via AAV‐mediated delivery. (A) Schematic diagram illustrating the experimental workflow of AAV‐mediated *Nat10* knockdown followed by cognitive function assessment; (B) RT‐qPCR analysis of *Nat10* mRNA expression in the hippocampal DG region; (C) WB analysis of NAT10 protein levels in the hippocampal DG region; (D, E) Immunofluorescence staining of NAT10 protein localization and expression in the DG region (scale bar: 25 μm); (F) EGFP immunofluorescence confirming accurate viral injection targeting the hippocampal DG region (scale bar: 25 μm). *n* = 6 animals per group. ns indicates no statistically significant difference; ***p < 0.001 between groups.

**Figure S2:** AAV‐mediated knockdown of *Nat10* in hippocampal DG neurons attenuates sevoflurane‐induced POCD in aged rats. (A, B) OFT assessing total locomotor distance and average speed; (C) Y‐maze test evaluating exploration time in the novel arm; (D) NORT measuring novel object exploration time and discrimination index; (E, F) MWM assessing escape latency during training and number of platform crossings during probe trials. Each group included 6 animals. “ns” indicates no significant difference; *p < 0.05, **p < 0.01, ***p < 0.001 vs. indicated group.

**Figure S3:** Single‐cell transcriptomic analysis of hippocampal tissue reveals sevoflurane‐induced alterations in cell cycle dynamics and transcriptional profiles. (A) Cell cycle scoring showing the distribution of S and G2/M phase scores (S.Score and G2M.Score) in Ctrl and Sevo groups; (B) Distribution of nFeature_RNA, nCount_RNA, and percent.mt across cells in each group; (C) Linear correlation analysis among nCount_RNA, nFeature_RNA, and percent.mt; (D) Highly variable gene analysis showing standardized variance versus mean expression; (E) Standard deviation plot for PCA; (F) Harmony‐based dimensional reduction displaying two‐dimensional distribution of integrated samples and embedding values of Harmony_1 across groups; (G) Dot plot of canonical marker gene expression across identified cell types. Each group included 3 animals.

**Figure S4:** Effect of *Sirt1* upregulation on GABABR1 expression via *Nat10*. (A) Schematic overview of the experimental procedure, including AAV viral injection and patch‐clamp recording; (B) immunofluorescence detection of EGFP signal confirming viral infection localization in the hippocampal DG region (scale bar: 25 μm); (C) WB analysis of SIRT1, NAT10, and GABABR1 protein expression in the hippocampal DG region; (D) RT‐qPCR quantification of *Gababr1* mRNA expression in the hippocampal DG region; (E) immunofluorescence analysis of GABABR1 protein localization and expression in the hippocampal DG region (scale bar: 25 μm). Each group included six animals. *p < 0.05, **p < 0.01, ***p < 0.001, compared between indicated groups.

**Figure S5:** Effect of SIRT1 Upregulation via NAT10 on GABABR1 Expression and Inhibitory Synaptic Currents. (A, B) Representative recordings of IPSCs in granule cells of the hippocampal DG from each group, acquired using whole‐cell patch‐clamp electrophysiology. Six animals were included per group. *Statistical significance between groups; ***p < 0.001.

**Figure S6:** Role of the *Sirt1*/*Nat10* axis in modulating autophagy, energy metabolism, and cognitive function. (A) Schematic illustration of the experimental protocol, including AAV injection and cognitive function assessments; (B) WB analysis of autophagy‐related proteins (LC3‐II/LC3‐I ratio and P62) in the hippocampal DG region; (C) ATP content in hippocampal tissue measured by colorimetric assay; (D) MMP assessed by JC‐1 staining; (E, F) Spontaneous alternation percentage measured by Y‐maze test; (G) Discrimination index evaluated via NORT; (H, I) Escape latency and platform crossings assessed by the MWM. Six animals were included per group. *p < 0.05, **p < 0.01, ***p < 0.001.

**Data S1:** cns70762‐sup‐0007‐Supinfo.docx.

## Data Availability

All data generated or analyzed during this study are included in this article and/or its Supporting Information. Further enquiries can be directed to the corresponding author.
