# Supplementary figures and images for: Targeting the SIRT1‐NAT10‐GABABR1 Axis: A Novel Epitranscriptomic Approach to Mitigate Sevoflurane‐Induced Cognitive Impairment in Aging
Source: CNS Neurosci Ther. 2026 Feb 4;32(2):e70762. doi: 10.1002/cns.70762 (PMC12869270; doi:10.1002/cns.70762)

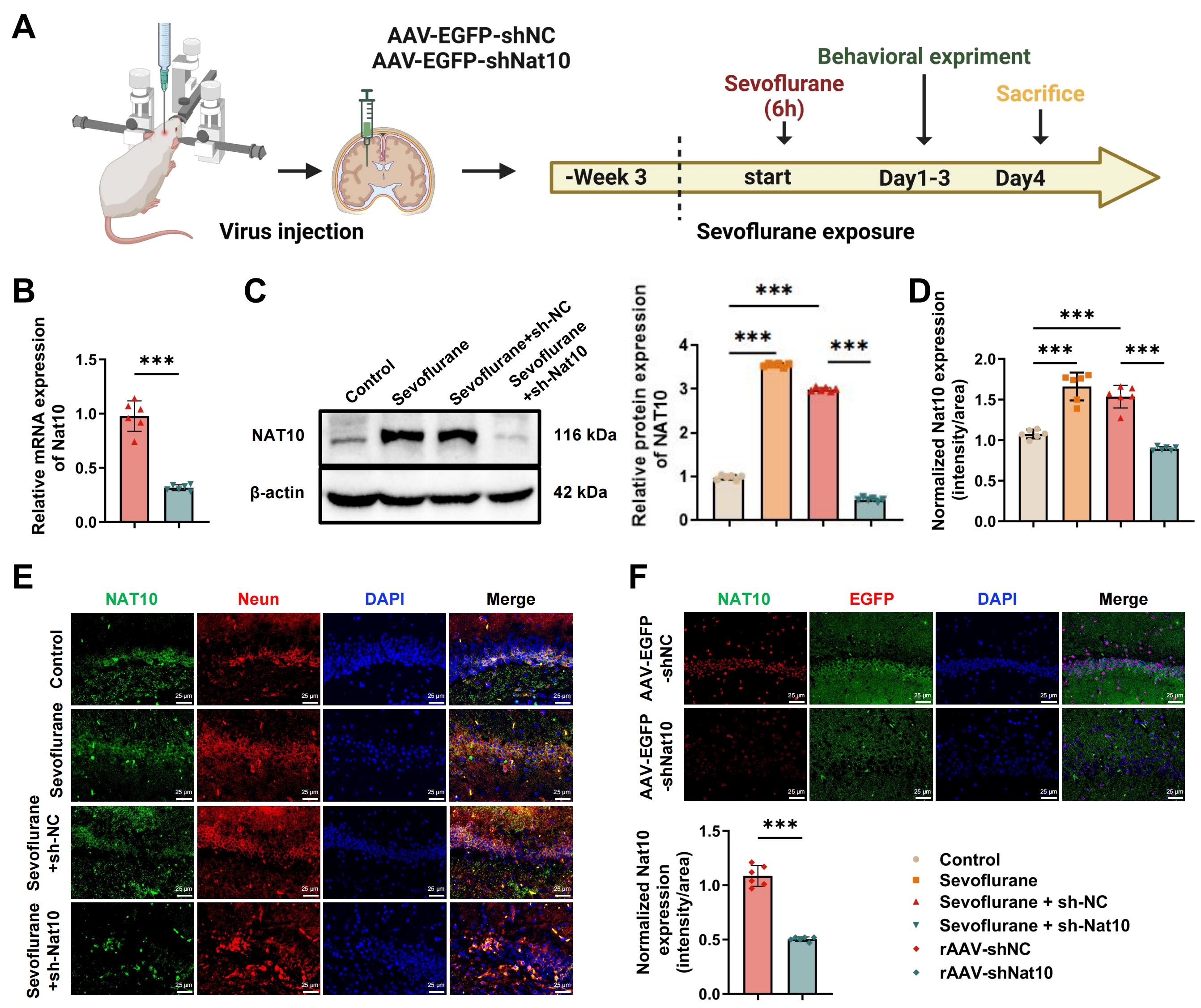

Supplement: Supplementary file 1 — Figure S1: Validation of Nat10 knockdown in hippocampal DG neurons via AAV‐mediated delivery. (A) Schematic diagram illustrating the experimental workflow of AAV‐mediated Nat10 knockdown followed by cognitive function assessment; (B) RT‐qPCR analysis of Nat10 mRNA expression in the hippocampal DG region; (C) WB analysis of NAT10 protein levels in the hippocampal DG region; (D, E) Immunofluorescence staining of NAT10 protein localization and expression in the DG region (scale bar: 25 μm); (F) EGFP immunofluorescence confirming accurate viral injection targeting the hippocampal DG region (scale bar: 25 μm). n = 6 animals per group. ns indicates no statistically significant difference; ***p < 0.001 between groups. [file CNS-32-e70762-s002.jpg]

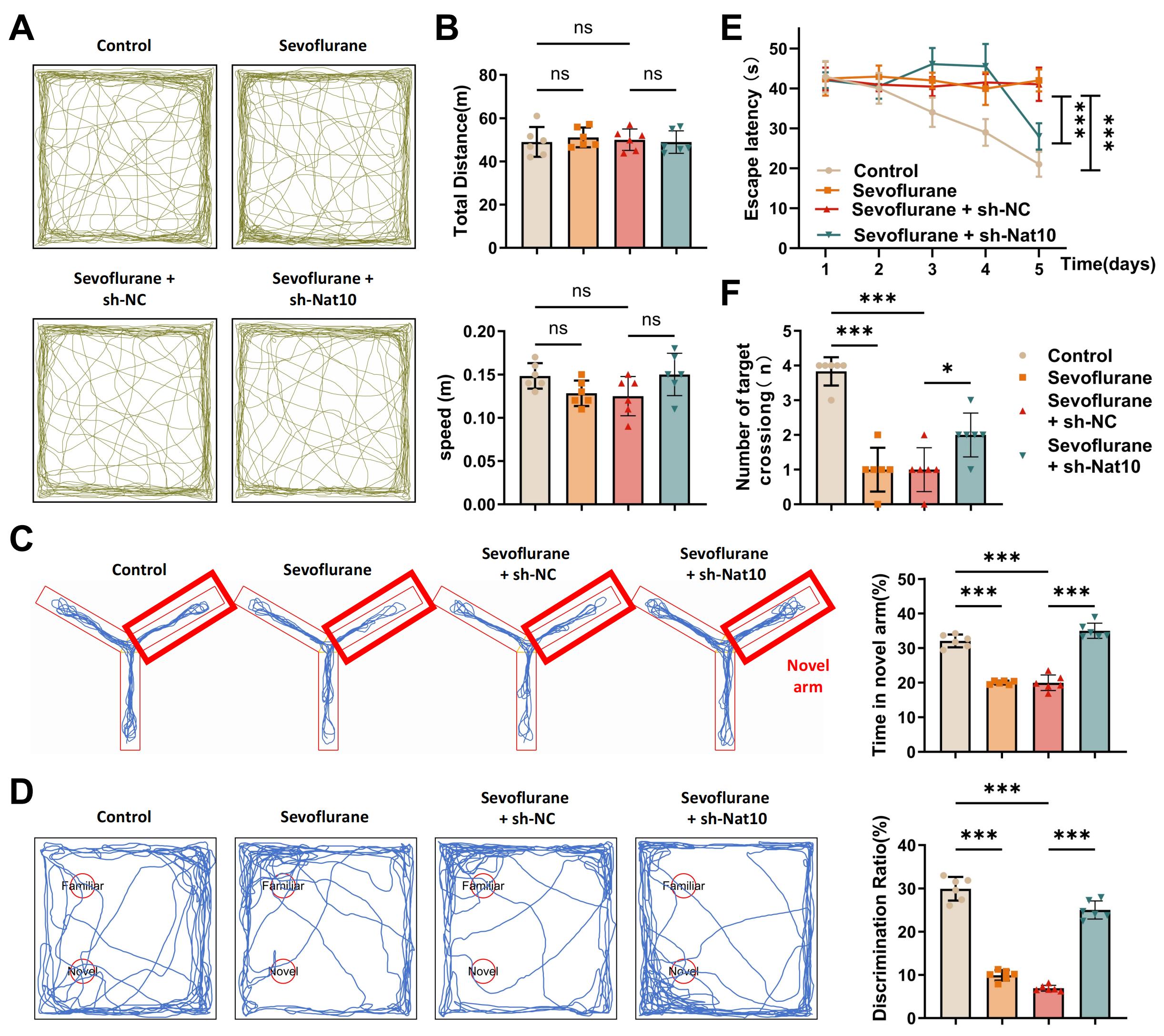

Supplement: Supplementary file 2 — Figure S2: AAV‐mediated knockdown of Nat10 in hippocampal DG neurons attenuates sevoflurane‐induced POCD in aged rats. (A, B) OFT assessing total locomotor distance and average speed; (C) Y‐maze test evaluating exploration time in the novel arm; (D) NORT measuring novel object exploration time and discrimination index; (E, F) MWM assessing escape latency during training and number of platform crossings during probe trials. Each group included 6 animals. “ns” indicates no significant difference; *p < 0.05, **p < 0.01, ***p < 0.001 vs. indicated group. [file CNS-32-e70762-s001.jpg]

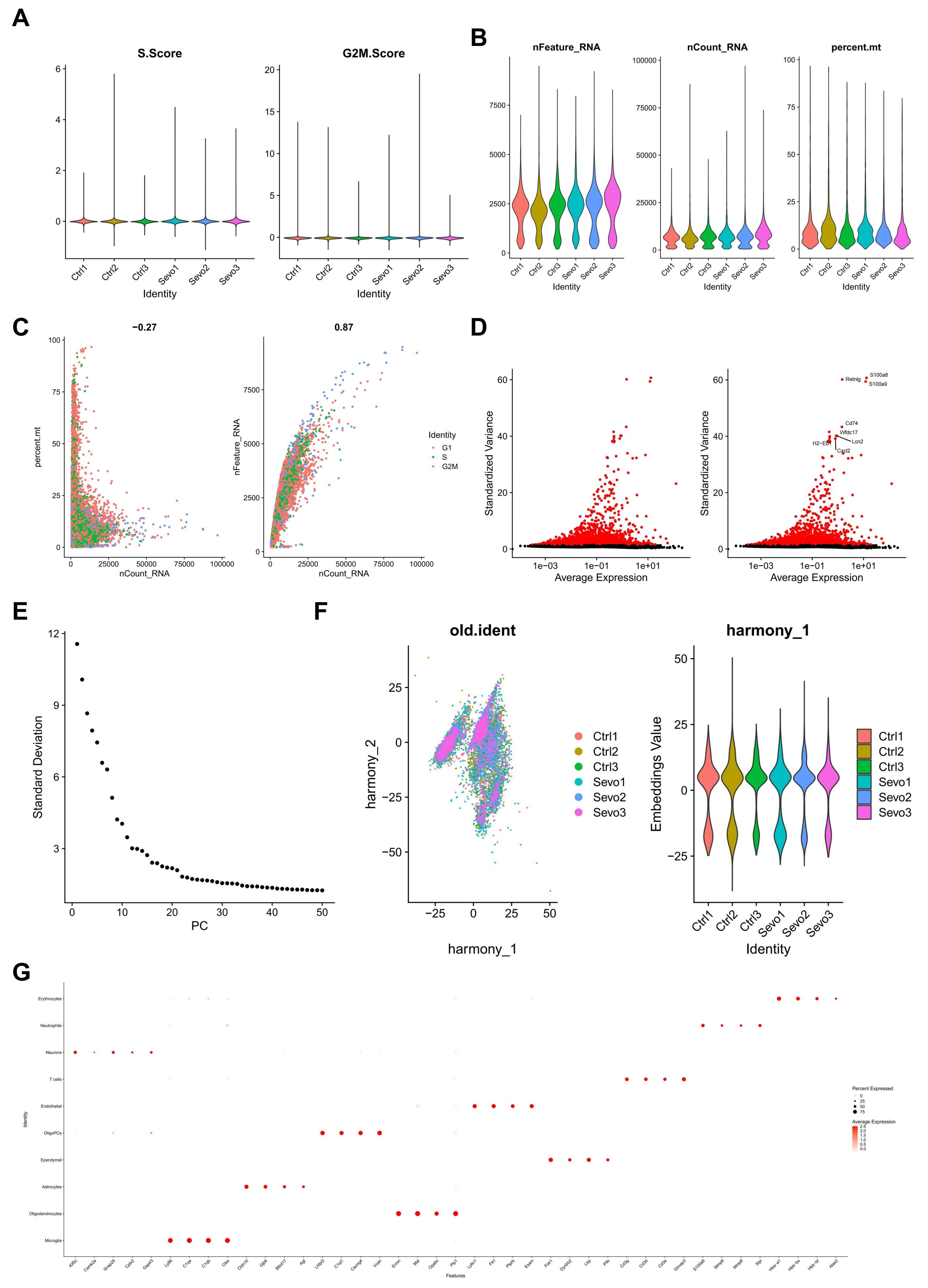

Supplement: Supplementary file 3 — Figure S3: Single‐cell transcriptomic analysis of hippocampal tissue reveals sevoflurane‐induced alterations in cell cycle dynamics and transcriptional profiles. (A) Cell cycle scoring showing the distribution of S and G2/M phase scores (S.Score and G2M.Score) in Ctrl and Sevo groups; (B) Distribution of nFeature_RNA, nCount_RNA, and percent.mt across cells in each group; (C) Linear correlation analysis among nCount_RNA, nFeature_RNA, and percent.mt; (D) Highly variable gene analysis showing standardized variance versus mean expression; (E) Standard deviation plot for PCA; (F) Harmony‐based dimensional reduction displaying two‐dimensional distribution of integrated samples and embedding values of Harmony_1 across groups; (G) Dot plot of canonical marker gene expression across identified cell types. Each group included 3 animals. [file CNS-32-e70762-s006.jpg]

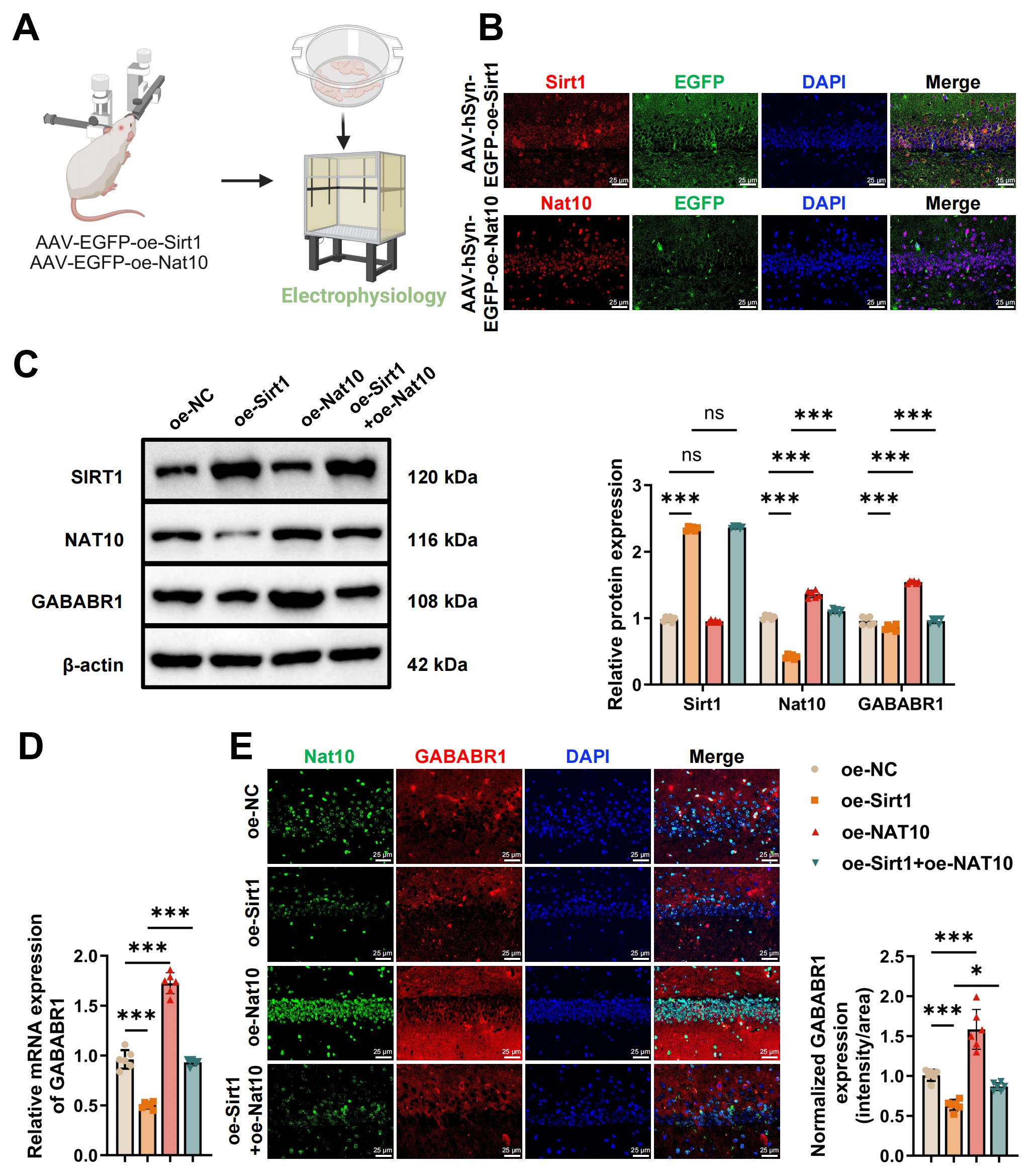

Supplement: Supplementary file 4 — Figure S4: Effect of Sirt1 upregulation on GABABR1 expression via Nat10. (A) Schematic overview of the experimental procedure, including AAV viral injection and patch‐clamp recording; (B) immunofluorescence detection of EGFP signal confirming viral infection localization in the hippocampal DG region (scale bar: 25 μm); (C) WB analysis of SIRT1, NAT10, and GABABR1 protein expression in the hippocampal DG region; (D) RT‐qPCR quantification of Gababr1 mRNA expression in the hippocampal DG region; (E) immunofluorescence analysis of GABABR1 protein localization and expression in the hippocampal DG region (scale bar: 25 μm). Each group included six animals. *p < 0.05, **p < 0.01, ***p < 0.001, compared between indicated groups. [file CNS-32-e70762-s005.jpg]

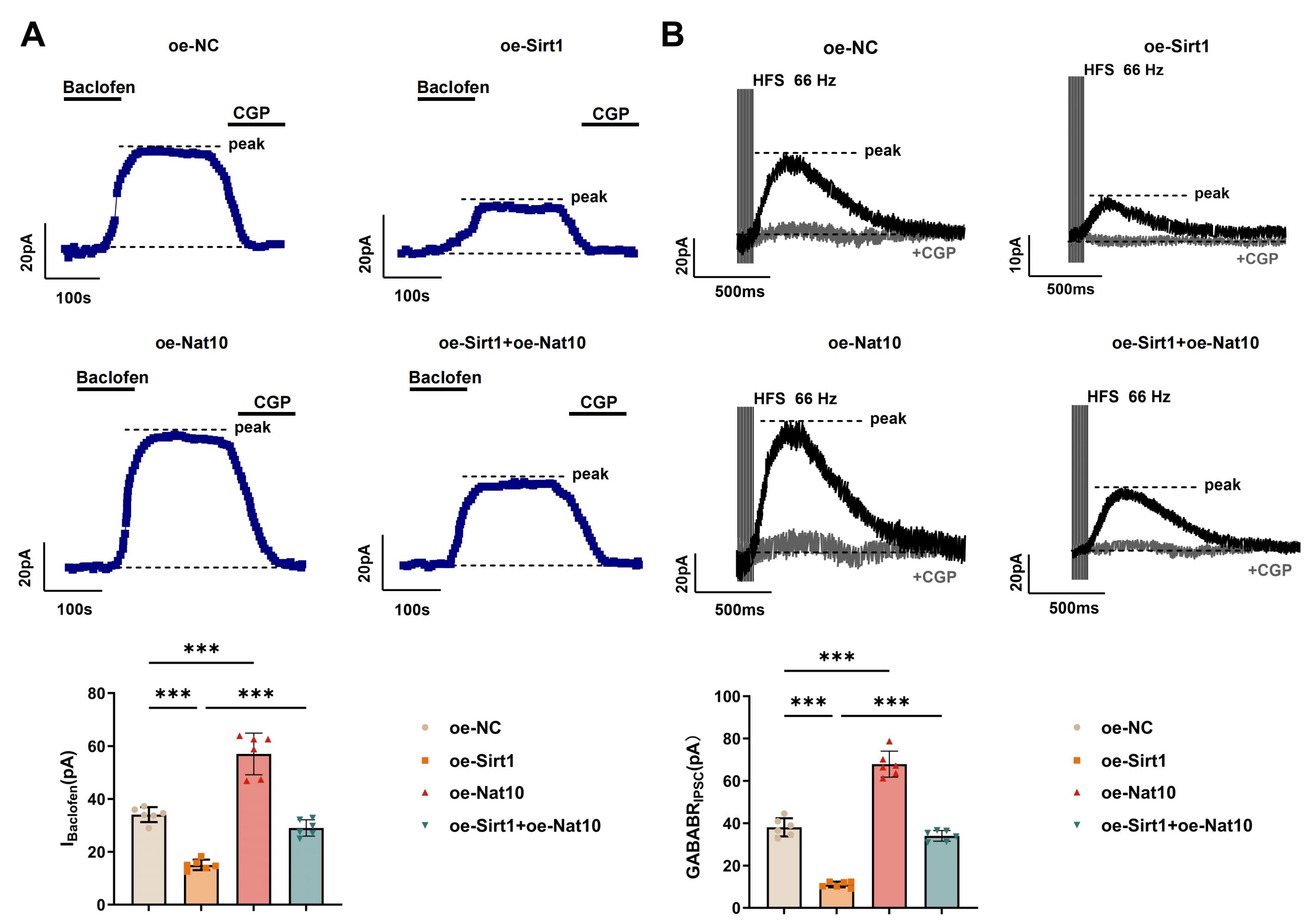

Supplement: Supplementary file 5 — Figure S5: Effect of SIRT1 Upregulation via NAT10 on GABABR1 Expression and Inhibitory Synaptic Currents. (A, B) Representative recordings of IPSCs in granule cells of the hippocampal DG from each group, acquired using whole‐cell patch‐clamp electrophysiology. Six animals were included per group. *Statistical significance between groups; ***p < 0.001. [file CNS-32-e70762-s003.jpg]

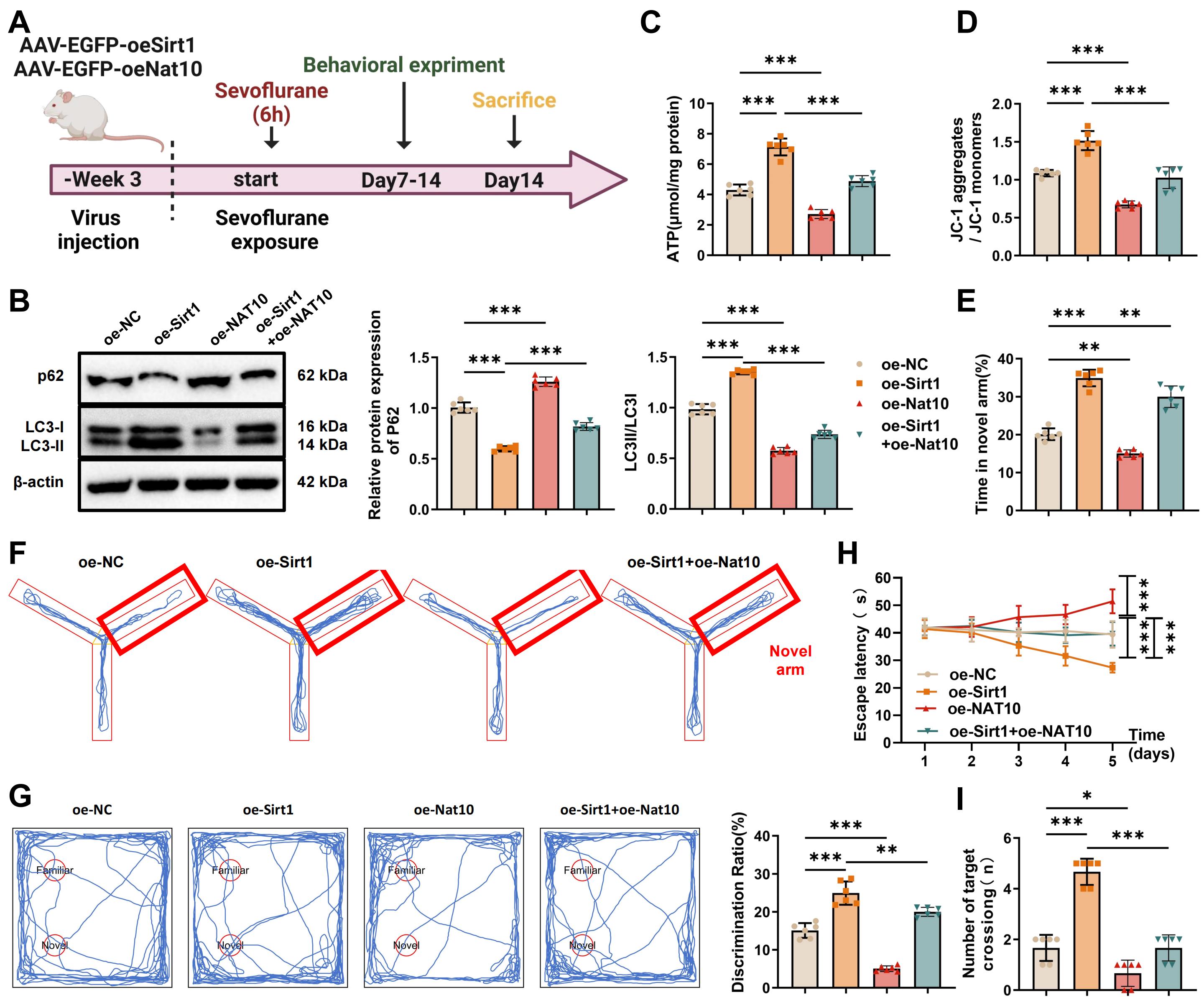

Supplement: Supplementary file 6 — Figure S6: Role of the Sirt1/Nat10 axis in modulating autophagy, energy metabolism, and cognitive function. (A) Schematic illustration of the experimental protocol, including AAV injection and cognitive function assessments; (B) WB analysis of autophagy‐related proteins (LC3‐II/LC3‐I ratio and P62) in the hippocampal DG region; (C) ATP content in hippocampal tissue measured by colorimetric assay; (D) MMP assessed by JC‐1 staining; (E, F) Spontaneous alternation percentage measured by Y‐maze test; (G) Discrimination index evaluated via NORT; (H, I) Escape latency and platform crossings assessed by the MWM. Six animals were included per group. *p < 0.05, **p < 0.01, ***p < 0.001. [file CNS-32-e70762-s004.jpg]
