# Supplementary material for: Targeting the SIRT1‐NAT10‐GABABR1 Axis: A Novel Epitranscriptomic Approach to Mitigate Sevoflurane‐Induced Cognitive Impairment in Aging
Source: CNS Neurosci Ther. 2026 Feb 4;32(2):e70762. doi: 10.1002/cns.70762 (PMC12869270; doi:10.1002/cns.70762)
Supplement: Supplementary file 7 — Data S1: cns70762‐sup‐0007‐Supinfo.docx. [file CNS-32-e70762-s007.docx]

Materials and Methods

**Preparation and Sequencing of Single-Cell Suspensions**

Fresh hippocampal tissue samples were collected from both the control group (n = 3) and the sevoflurane-treated group (n = 3) of Sprague-Dawley (SD) rats. Tissues were enzymatically dissociated using the Papain Dissociation System (Worthington Biochemical Corporation, USA) followed by mechanical trituration to obtain single-cell suspensions. Cell viability was assessed using the Cellometer Auto 2000 (Nexcelom Bioscience, USA), and only suspensions with viability >85% were used for downstream processing. Single-cell suspensions were processed using the 10× Genomics Chromium platform (10× Genomics, USA) for cell barcoding and library construction. Sequencing was performed on the Illumina NovaSeq 6000 system (Illumina, USA) using paired-end 150 bp reads. Raw sequencing data were processed with Cell Ranger (v6.1.2, 10× Genomics) to generate FASTQ files, align reads to the Rattus norvegicus reference genome (Rnor_6.0), and count gene transcripts. Quality control was conducted using the Seurat R package (v4.3.0), with cells excluded if they exhibited >15% mitochondrial gene content or fewer than 200 detected genes. High-quality cells were retained for downstream analysis, and mitochondrial content, median gene counts, and UMI distributions were comparable across groups.

**Dimensionality Reduction, Clustering, and Cell Type Annotation**

Normalization of expression data was performed using the SCTransform function in Seurat, followed by sample integration via the FindIntegrationAnchors and IntegrateData functions. Batch effects were corrected using the Harmony package (v0.1.1), and dimensionality reduction was conducted using principal component analysis (PCA). Clustering was performed based on the top 30 principal components at a resolution of 0.6, with two-dimensional visualization achieved using Uniform Manifold Approximation and Projection (UMAP). Marker genes for each cluster were identified using the FindAllMarkers function (min.pct = 0.25, logfc.threshold = 0.25), and cell types were annotated with reference to published literature and the CellMarker database ([http://biocc.hrbmu.edu.cn/CellMarker/](http://biocc.hrbmu.edu.cn/CellMarker/)).

**Autophagy Scoring and Subpopulation Classification**

Autophagy-related gene sets were obtained from the MSigDB database (HALLMARK_AUTOPHAGY, [https://www.gsea-msigdb.org/](https://www.gsea-msigdb.org/)). The AddModuleScore function in Seurat was used to compute autophagy scores for neuronal populations. Based on these scores, neurons were classified into high-autophagy and low-autophagy subpopulations.

**Differential Expression and Functional Enrichment Analysis**

Differential gene expression analysis between high and low autophagy-scoring neurons was performed using the FindMarkers function with parameters logfc.threshold = 0.25 and min.pct = 0.1. Genes with |log₂FC| > 0.25 and *p* < 0.05 were considered significantly differentially expressed. Notably, genes such as Sirt1 were significantly enriched in neurons with high autophagy scores and were selected for downstream functional annotation. The differentially expressed genes (DEGs) were subjected to Gene Ontology (GO) and Kyoto Encyclopedia of Genes and Genomes (KEGG) enrichment analyses using the clusterProfiler package (v4.6.2), with significance defined as adjusted *p* < 0.05 (Benjamini–Hochberg correction). Gene ID conversion was performed based on the org.Rn.eg.db database.

**Establishment of the POCD Model in Aged Rats**

Eighteen-month-old specific-pathogen-free (SPF) male Sprague-Dawley (SD) rats (550–600 g, strain code: 101; Beijing Vital River Laboratory Animal Technology Co., Ltd., China) were housed under controlled conditions (temperature: 22 ± 2°C, humidity: 50%–60%, 12-hour light/dark cycle) with ad libitum access to standard chow and water. Rats were randomly assigned to eight groups (n = 10 per group): Control, Sevoflurane, sh-NC, sh-Nat10, oe-NC, oe-Sirt1, oe-Nat10, and oe-Sirt1+oe-Nat10. Sevoflurane-exposed groups were placed in an animal anesthesia chamber containing 2% sevoflurane for 5 hours. The chamber temperature was maintained at 37 ± 1°C using an infrared heat lamp. All animal procedures were approved by the Institutional Animal Care and Use Committee.

**AAV Vector Construction and Stereotactic Injection**

Recombinant AAV vectors were constructed according to experimental requirements, including rAAV-human synapsin promoter (hSyn)-EGFP-sh-Nat10, rAAV-hSyn-EGFP-sh-NC, rAAV-hSyn-EGFP-oe-Sirt1, rAAV-hSyn-EGFP-oe-Nat10, rAAV-hSyn-EGFP-oe-Sirt1+oe-Nat10, and a control vector rAAV-hSyn-EGFP-oe-NC (Braincase Co., Ltd., Wuhan, China). All vectors had a titer of 1×10¹² vg/mL. Under anesthesia, rats were secured in a stereotaxic apparatus (RWD Life Science, China), and 1 μL of virus was bilaterally injected into the dentate gyrus (DG) of the hippocampus using the following coordinates: AP: –3.6 mm, ML: ±2.5 mm, DV: –3.5 mm. Injections were administered at a rate of 0.5 μL/min, and the needle was retained in place for 5 minutes post-injection. Animals were maintained for three weeks post-surgery to allow sufficient viral expression. To verify the efficiency and tissue specificity of viral expression, RT-qPCR was performed to assess the mRNA expression level of Nat10, confirming both the viral transduction efficiency and knockdown effect. Western blot analysis was used to examine changes in the expression of the target protein Nat10 within the DG region, thereby validating effective viral expression in this area. In addition, immunofluorescence staining was conducted to detect the EGFP signal, confirming the precise localization of the virus in the hippocampal DG region.

**WB Analysis of Protein Expression**

Hippocampal DG tissues or primary hippocampal neurons from each group were lysed using RIPA buffer (P0013B, Beyotime, China), and total protein concentrations were determined with a BCA Protein Assay Kit (P0012, Beyotime, China). Equal amounts of protein (30 μg per sample) were separated by SDS-PAGE and subsequently transferred to polyvinylidene difluoride (PVDF) membranes (IPVH00010, Millipore, USA). Membranes were blocked with 5% non-fat milk for 1 hour at room temperature and incubated overnight at 4 °C with the following primary antibodies: anti-NAT10 (1:2000, ab194297, Abcam, UK), anti-SIRT1 (1:1000, ab110304, Abcam, UK), anti-GABABR1 (1:1000, ab55051, Abcam, UK), anti-LC3B (1:1000, 3868, Cell Signaling Technology, USA), anti-P62 (1:1000, ab109012, Abcam, UK), anti-acetyl-lysine (1:1000, #6952, Cell Signaling Technology, USA), and anti-β-actin (1:1000, ab8226, Abcam, UK). After washing with PBST, membranes were incubated for 1 hour with HRP-conjugated secondary antibodies: Goat Anti-Rabbit IgG H&L (ab6721) or Rabbit Anti-Mouse IgG H&L (ab6728, both from Abcam, UK). Protein bands were visualized using an enhanced chemiluminescence detection system (WBKLS0500, Millipore, USA). Band intensities were quantified using ImageJ software (NIH, USA), and all target protein levels were normalized to β-actin.

**RT-qPCR Analysis of mRNA Expression**

Total RNA was isolated from the DG region of the hippocampus using TRIzol reagent (15596026, Invitrogen, USA), and RNA purity was assessed via NanoDrop 2000 spectrophotometry (Thermo Fisher Scientific, USA). cDNA synthesis was performed using the PrimeScript RT Reagent Kit (RR037A, Takara, Japan), and quantitative PCR was carried out with SYBR Green Master Mix (A25742, Thermo Fisher Scientific, USA) on an ABI 7500 real-time PCR system (Applied Biosystems, USA). β-actin was used as the internal control, and relative gene expression was calculated using the 2⁻ΔΔCT method. Primer sequences used were as follows: *Nat10*: Forward 5′-CGTGCATTCATTGTGCGACT-3′; Reverse 5′-AAAAGAGACCTCTGCCGCTC-3′; *GABABR1*: Forward 5′-TAGAGCCGTGAACACCCAAC-3′; Reverse 5′-AGGTGCTGAGTCATACTGTTCA-3′; *β-actin*: Forward 5′-CCCGCGAGTACAACCTTCTT-3′; Reverse 5′-AACACAGCCTGGATGGCTAC-3′

**Immunohistochemical Detection of NAT10 Expression**

Rat hippocampal tissues were fixed in 4% paraformaldehyde (P0099, Beyotime, China) for 24 hours, dehydrated, embedded in paraffin, and sectioned at a thickness of 4 μm. Endogenous peroxidase activity was quenched using 3% hydrogen peroxide, followed by blocking with 5% bovine serum albumin (BSA; A1933, Sigma-Aldrich, USA) for 1 hour at room temperature. Sections were incubated overnight at 4°C with a rabbit anti-NAT0 antibody (1:500, ab194297, Abcam, UK). The next day, HRP-conjugated goat anti-rabbit IgG H&L (ab6721, Abcam, UK) was applied, and immunoreactivity was visualized using DAB chromogen (ZLI-9018, ZSGB-Bio, China). Counterstaining was performed with hematoxylin. Images were acquired using an Olympus BX53 microscope (Japan), and the average optical density was quantified using Image-Pro Plus 6.0.

**Detection of mRNA ac⁴C Modification**

Total RNA was extracted from rat hippocampal tissue, and mRNA was isolated using the Dynabeads® mRNA Purification Kit (61006D, Thermo Fisher Scientific, USA). Equal amounts of mRNA were denatured at 65°C for 5 minutes and then spotted onto a positively charged nylon membrane (GE Healthcare, USA). The membrane was UV-crosslinked for 30 minutes, blocked with 5% non-fat milk at room temperature for 1 hour, and incubated overnight at 4°C with an anti-ac⁴C-specific antibody (1:200, ab252215, Abcam, UK). On the following day, an HRP-conjugated goat anti-rabbit secondary antibody (ZB-2301, ZSGB-Bio, China) was applied for 1 hour. Signal detection was performed using enhanced chemiluminescence (ECL; WBKLS0500, Millipore, USA). Methylene blue staining was used to verify equal RNA loading.

**Immunofluorescence Staining**

Rats were deeply anesthetized with isoflurane and perfused transcardially with 4% paraformaldehyde (P0099, Beyotime, China) to fix brain tissues. Following fixation, brains were dehydrated in a graded sucrose solution (10%, 20%, and 30%), and 30 μm-thick coronal sections were prepared using a cryostat (CM1520, Leica, Germany). The sections were incubated in a humid chamber at 4°C for 12 hours with the following primary antibodies: rabbit anti-NAT10 (1:200, ab194297, Abcam, UK), rabbit anti-SIRT1 (1:100, ab189494, Abcam, UK), rabbit anti-GFAP (1:500, 3670, Cell Signaling Technology, USA), mouse anti-NEUN (1:500, MAB377, Millipore, USA), mouse anti-IBA-1 (1:400, SAB2702364, Millipore, USA), mouse anti-GFAP (1:500, ab7260, Abcam, UK), mouse anti-CaMKIIα (1:100, 50049, Cell Signaling Technology, USA), chicken anti-GAD67 (1:1000, ab75712, Abcam, UK), and mouse anti-GABABR1 (1:200, ab55051, Abcam, UK). After washing with Phosphate-Buffered Saline (PBS), sections were incubated for 1 hour at room temperature with the appropriate secondary antibodies: goat anti-rabbit Alexa Fluor 488 (ab150077), goat anti-rabbit Alexa Fluor 647 (ab170079), goat anti-mouse Alexa Fluor 647 (ab150115), and goat anti-chicken Alexa Fluor 594 (ab150172) (all from Abcam, UK). Nuclei were counterstained with 4',6-diamidino-2-phenylindole (DAPI) (C1005, Beyotime, China) for 5 minutes. Sections were washed, mounted with an antifade mounting medium, and imaged using a fluorescence microscope (BX53, Olympus, Japan).

**ATP Quantification**

ATP levels were assessed using both luciferase-based luminescence and colorimetric assays. For animal experiments, hippocampal tissues were rapidly harvested post-treatment. Mitochondrial fractions were isolated and lysed in RIPA buffer (P0013B, Beyotime, China), and protein concentrations were determined using a BCA assay (P0010, Beyotime, China). A 50 μL aliquot of each lysate was incubated with the ATP detection reagent (Shanghai Tianhua Biotechnology Co., China), and luminescence was measured at room temperature. All sample preparations, reagent handling, and loading were conducted on ice (0°C), with centrifugation steps performed at 4°C to preserve ATP stability. Luminescent signals were recorded using a microplate reader (SpectraMax iD5, Molecular Devices, USA), and ATP concentrations were calculated based on a standard curve.

For cell-based assays, primary neurons were cultured under the indicated conditions for 48 hours. Cells were then lysed in RIPA buffer and centrifuged at 12,000 rpm for 10 minutes at 4°C. The supernatants were used for ATP quantification with a colorimetric ATP assay kit (S0026, Beyotime, China). Absorbance was measured using a microplate spectrophotometer (SpectraMax iD5), and ATP levels were determined according to the corresponding standard curve.

**Assessment of MMP via JC-1 Staining**

In animal experiments, mitochondria were isolated from rat hippocampal tissue using a Mitochondria Extraction Kit (Solarbio, Beijing, China). Tissue samples were homogenized on ice and centrifuged at 4°C to collect the mitochondrial fraction, which was stored at –70°C until further use. Protein concentrations were quantified using the BCA assay (P0010, Beyotime, China), and all samples were diluted to equivalent concentrations. JC-1 working solution was prepared according to the manufacturer’s instructions (C2006, Beyotime, China) and incubated with the mitochondrial preparations under sterile conditions on ice. Fluorescence intensities were measured at excitation/emission wavelengths of 525/590 nm for JC-1 aggregates and 490/530 nm for monomers. The red-to-green fluorescence ratio was calculated to assess changes in MMP.

In cellular assays, neurons from each experimental group were stained with JC-1 dye (C2006, Beyotime, China) and incubated at 37°C for 30 minutes. After rinsing with PBS, fluorescence was immediately analyzed using a BD FACSCanto II flow cytometer (BD Biosciences, USA) to determine the red/green ratio as an indicator of MMP.

**Open Field Test (OFT)**

The open field apparatus consisted of a square chamber (50 cm × 50 cm × 40 cm) subdivided into four equal quadrants marked on the floor. Rats were individually placed in the center of the arena and allowed a 30-second acclimation period. Each animal was then observed for 10 minutes, during which its movement was recorded using a video camera positioned 120 cm above the field. Total distance traveled and average velocity were measured. The test was conducted under standard illumination conditions (800 lux). All video data analyses, including the calculation of exploration time and movement speed, were performed under double-blind conditions. Data processing was conducted by investigators who were unaware of the experimental group assignments to minimize subjective bias.

**Y-Maze Test**

To assess spatial working memory in rats, the Y-maze behavioral test was employed. The apparatus consists of three arms of equal length (designated A, B, and C) arranged at 120° angles to form a Y-shaped configuration. During the training phase, one arm was randomly closed while the other two remained accessible. Rats were placed in the central zone and allowed to freely explore the two open arms for 5 minutes. After a 2-hour retention interval, the test phase commenced: the previously closed arm was opened, and all three arms were made accessible. Rats were reintroduced to the maze and permitted to explore freely for 3 minutes. The duration spent in the newly opened arm was recorded to evaluate spatial recognition and memory performance. All video data analyses, including the calculation of exploration time, were performed under double-blind conditions to ensure that data analysts were unaware of the experimental group assignments, thereby avoiding subjective bias.

**NORT**

On the first day, rats were placed in an empty testing chamber for 10 minutes to allow habituation to the environment. On the second day, during the training session, two identical objects (matched in shape and size) were positioned in opposite corners of the chamber. Rats were then allowed to explore freely for 10 minutes. Following a 2-hour delay, the testing phase was conducted by replacing one of the original objects with a novel item of similar size but different shape. Rats were reintroduced into the chamber and permitted to explore for 5 minutes. The time spent exploring the novel object (T_novel) and the familiar object (T_familiar) was recorded. The discrimination index (DI) was calculated as follows:

DI = T _novel_ / (T _nove_l + T _familiar_) × 100%

This index quantifies recognition memory by reflecting the animal’s preference for novelty. All video data analyses, including the calculation of exploration time and discrimination index, were performed under double-blind conditions to ensure that the investigators were unaware of the experimental group assignments of the rats.

**MWM**

The MWM was employed to assess spatial memory performance. Each group of six rats underwent training across four quadrants for five consecutive days, with each trial lasting 60 seconds. Rats were introduced sequentially from a fixed point in each quadrant, and the latency to locate the hidden platform was recorded as the escape latency. Upon locating the platform, the rats were allowed to remain on it for 15 seconds. If a rat failed to find the platform within 60 seconds, it was gently guided to it and allowed to stay for 15 seconds. The average escape latency for each quadrant was calculated daily. On the fifth day, a spatial probe test was conducted by removing the platform and allowing the rats to freely explore the maze for 60 seconds. The time spent in the target quadrant was recorded as a measure of spatial memory retention. All video data analyses, including the calculation of latency and dwell time, were performed under double-blind conditions to ensure that data analysts were blinded to the group assignments of the rats, thereby minimizing potential subjective bias.

**Brain Slice Preparation and Electrophysiological Recording**

Coronal hippocampal slices (300 µm thickness) were prepared using a vibrating microtome (Leica VT1200) and incubated in oxygenated artificial cerebrospinal fluid (ACSF). The ACSF had a pH of 7.3–7.4 and an osmolarity of 300–305 mOsm, with the following composition (in mM): 124 NaCl, 2.5 KCl, 2 MgSO₄, 2 CaCl₂, 1.25 NaH₂PO₄, 26 NaHCO₃, 11 D-glucose, and 2 MgCl₂. Slices were incubated at 34°C for 30 minutes and then maintained at room temperature for an additional hour before being transferred to a perfusion chamber mounted on an upright microscope (Olympus BW51). Recordings were performed in continuously perfused ACSF. Patch electrodes were pulled using a Sutter P97 puller from borosilicate glass capillaries (outer diameter: 1.5 mm; inner diameter: 0.86 mm; Sutter BF150-86-10), yielding a resistance of 3–8 MΩ. The internal solution used for whole-cell recordings contained (in mM): 110 potassium gluconate, 30 KCl, 10 Na-phosphocreatine, 10 HEPES, 4 MgATP, 0.3 Na₃GTP, and 0.3 EGTA, adjusted to pH 7.3 with KOH and an osmolarity of 290 mOsm.

To evaluate the effects of the GABA_B receptor agonist Baclofen (300 μM; Millipore, USA) and the selective antagonist CGP52432 (100 μM; Millipore, USA), recordings were conducted using the aforementioned internal solution. Voltage values were not corrected for liquid junction potential. Series resistance was compensated in current-clamp mode and maintained below 30 MΩ. Signals were sampled at 10 kHz and filtered at 2 kHz using a Digidata 1550A system (Molecular Devices). Data were acquired using a Multiclamp 700B amplifier and analyzed with pClamp 10.7 software (Molecular Devices). To measure slow inhibitory postsynaptic currents (IPSC_slow) mediated by GABA_B receptors, a concentric bipolar stimulating electrode (FHC, Bowdoinham, ME) was positioned near the recording site. Electrical stimulation was delivered via an ISO-flex isolated pulse stimulator (A.M.P.I., Jerusalem, Israel). Synaptic currents were evoked by brief 66 Hz trains of 10 pulses under a voltage clamp at –60 mV in the presence of picrotoxin, NBQX, and D-APV to block GABA_A and glutamatergic transmission.

**Primary Hippocampal Neuron Culture and Experimental Grouping**

Hippocampal tissues were harvested from embryonic day 18 (E18) SPF Sprague-Dawley rat embryos (Beijing Vital River Laboratory Animal Technology Co., Ltd., China). The tissue was enzymatically dissociated using 0.125% trypsin (25200056, Gibco, USA) at 37 °C for 15 minutes, followed by gentle mechanical trituration and centrifugation to isolate primary hippocampal neurons. Cells were seeded at a density of 1×10⁶ cells/mL onto 6-well plates (Corning, USA) pre-coated with poly-D-lysine (0.1 mg/mL, Sigma, USA). Neurons were cultured in Neurobasal medium (21103-049, Gibco, USA) supplemented with 2% B27 (A3582801, Gibco, USA) and 0.25% GlutaMAX (35050061, Gibco, USA). On day 3 *in vitro*, Cytosine arabinoside (Ara-C) (2.5 µg/mL, C1768, Sigma, USA) was added for 24 hours to inhibit glial proliferation. Thereafter, half of the culture medium was refreshed every three days, and neurons were maintained for 14 days.

Prior to viral transduction, neurons were exposed to a gas mixture containing 4.1% sevoflurane (with 5% CO₂ and 90.9% O₂) for 4 hours using a closed-circuit gas delivery system at a flow rate of 2 L/min. Eight experimental groups were established: Control, Sevoflurane, Sevoflurane + sh-NC, Sevoflurane + sh-Nat10, oe-NC, oe-Sirt1, oe-Nat10, and oe-Sirt1 + oe-Nat10. After sevoflurane exposure, cells were transferred to 6-well plates (1×10⁶ cells/well), incubated at 37 °C for 24 hours, and subsequently subjected to viral transfection. Cells were harvested 72 hours post-transfection for further analysis.

**Viral Transfection**

Primary hippocampal neurons were seeded in 6-well plates containing serum-free medium at a density of 1×10⁶ cells per well. Cells were treated with 1 mL of the corresponding viral vectors: sh-NC, sh-Nat10, oe-NC, oe-Sirt1, oe-Nat10, or oe-Sirt1 + oe-Nat10 (GenePharma, China). Transfection was carried out using Lipofectamine 3000 reagent (13000001, Thermo Fisher Scientific, USA), following the manufacturer's instructions. Post-transfection, neurons were maintained in a Neurobasal medium at 37 °C in a 5% CO₂ incubator for 48 hours. RT-qPCR was performed to confirm transfection efficiency and gene expression levels. The shRNA sequences were as follows: *Nat10* shRNA-1: ACAAGAAAGAGCTGGGATTTA; *Nat10* shRNA-2: AGAGTGGGACCTTGAACATAA; sh-NC (negative control): TTCTCCGAACGTGTCACGT.

**Immunofluorescence Detection of Green Fluorescent Protein (GFP)-LC3 Puncta Formation**

When cell confluency reached approximately 70%, neurons were transfected with GFP-LC3 plasmids (Vigene Biosciences, China) using Lipofectamine 3000 (L3000015, Invitrogen, USA). After 18 hours, cells were fixed in 4% paraformaldehyde (P0099, Beyotime, China) for 15 minutes and washed three times with PBS. Permeabilization was performed using 0.3% Triton X-100 (T8787, Sigma-Aldrich, USA) for 10 minutes at room temperature, followed by blocking with 1% BSA (A8020, Solarbio, China) for 1 hour. Nuclear staining was conducted using DAPI (C1005, Beyotime, China) for 5 minutes in the dark at room temperature. After an additional three PBS washes, images were acquired using an LSM 880 confocal microscope (Zeiss, Germany). The proportion of cells exhibiting GFP-LC3 puncta was quantified among 100 GFP-LC3–positive cells per group to evaluate autophagosome formation.

**Immunofluorescence Co-localization of LC3 and TOM20**

Cells were fixed in 4% paraformaldehyde for 15 minutes and washed three times with PBS. Permeabilization was carried out with 0.3% Triton X-100 for 10 minutes at room temperature. After blocking with 5% BSA for 1 hour, cells were incubated overnight at 4 °C with rabbit anti-LC3 (1:100, VB2930-50, Amyjet Scientific, China) and mouse anti-TOMM20 (1:100, ab186735, Abcam, UK) antibodies. After PBS washes, Alexa Fluor 488– and Alexa Fluor 594–conjugated secondary antibodies (1:200, Amyjet Scientific, China) were applied for 2 hours in the dark. Nuclei were counterstained with DAPI for 5 minutes. Fluorescent images were captured using the LSM 880 confocal microscope, and LC3-TOM20 co-localization was quantified using ImageJ software.

**Measurement of Intracellular ROS Levels**

Primary neurons from each group were incubated with 10 μM DCFH-DA (S0033S, Beyotime, China) in a serum-free medium for 30 minutes in the dark. Cells were then washed three times with PBS to remove excess dye. Intracellular fluorescence intensity was measured using a BD FACSCanto II flow cytometer (BD Biosciences, USA) through the PE-Texas Red channel (Ex/Em = 488/525 nm). A total of 10,000 cells were analyzed per group, and the mean fluorescence intensity (MFI) was calculated using FlowJo software to quantify ROS levels.

**CCK-8 Assay for Cell Viability**

Primary hippocampal neurons derived from neonatal mice were seeded into 96-well plates at a density of 5 × 10³ cells per well in 100 μL of serum-free medium. After incubation at 37 °C with 5% CO₂ for 24 hours, cells were treated with 2 mM 2-deoxy-D-glucose (2-DG; D8375, Sigma-Aldrich, USA) for 2 hours. Subsequently, 10 μL of CCK-8 reagent (abs50003, Absin, China) was added to each well and incubated for an additional 2 hours at 37 °C. Absorbance at 450 nm was measured using a microplate reader (Biotek, Winooski, VT, USA). Relative cell viability was calculated as the ratio of the absorbance of experimental wells to that of blank control wells (cell viability = OD_sample / OD_blank × 100%).

**Plasmid Construction and Mutagenesis**

To investigate protein function and interactions, various expression plasmids were constructed. Full-length *Nat10* and its truncated mutants—including the N-terminal, HAT domain, and C-terminal regions—were cloned into either pCI-neo or pEGFP-C2 vectors with Flag or GFP tags. Similarly, Flag-tagged constructs of *Sirt1*, *Sirt6*, *Sirt7*, and *Che-1* were cloned into pCI-neo for overexpression studies. For *in vitro* binding assays, GST-tagged versions of *Nat10* and *Sirt1* were subcloned into the pGEX-4T1 vector for subsequent GST pull-down experiments. All constructs containing PCR-amplified inserts were validated by Sanger sequencing to confirm the correct insertion.

Truncation mutants of *Nat10* and the catalytically inactive mutant of *Sirt1* (HA-Sirt1 H363Y) were generated using the QuickChange Site-Directed Mutagenesis Kit (Stratagene), following the manufacturer’s protocol.

**Co-Immunoprecipitation (Co-IP) Assay**

Primary hippocampal neurons were lysed using RIPA buffer (P0013B, Beyotime, China) for initial protein extraction, followed by further lysis in Buffer A (25 mM Tris-Cl, pH 7.5; 150 mM KCl; 1 mM DTT; 2 mM EDTA; 0.5 mM PMSF; 0.2% Nonidet P-40). Protein concentrations were quantified using a BCA assay (P0012, Beyotime, China). Equal amounts of total protein from each sample were incubated with Protein A agarose beads (GE Healthcare) pre-conjugated with specific primary antibodies. Antibody binding was achieved by incubating the beads in a 50% slurry with antibody solution (IPP500 buffer: 500 mM NaCl, 10 mM Tris-Cl, pH 8.0, 0.2% Nonidet P-40) at 4 °C for 1 hour. Immunoprecipitation was carried out by incubating the antibody-bound beads with the protein lysates at 4 °C for 2 hours with rotation. Beads were then washed three times (5 min each) with IPP500 buffer to remove non-specific interactions. The bound complexes were eluted by boiling in SDS sample buffer and analyzed by WB to detect the target proteins.

***In Vitro* Deacetylation Assay**

Purified His-tagged NAT10 protein bound to Ni-NTA beads was first incubated in acetylation buffer containing 50 mM Tris-Cl (pH 7.9), 10% glycerol, 0.1 mM EDTA, 1 mM PMSF, 10 mM sodium butyrate, and 10 μM acetyl-CoA (Sigma-Aldrich, USA) at 30 °C for 1 hour to achieve acetylation *in vitro*. After incubation, the beads were washed three times with the same buffer (5 min per wash) to remove excess acetyl-CoA. The acetylated His-Nat10 was then subjected to deacetylation assays by incubation with 0.25–1.0 μg of acetylated His-Nat10 and 0.5 μg of Flag-tagged Sirt1 in reaction buffer (50 mM Tris-Cl, pH 7.5), in the presence or absence of 1 mM NAD⁺ (Sigma-Aldrich, USA) at 30 °C for 1 hour. Samples were boiled, resolved by SDS-PAGE, transferred to PVDF membranes (IPVH00010, Millipore, USA), and probed for acetylation using an anti-acetyl-lysine antibody (1:1000, #6952, Cell Signaling Technology, USA), followed by HRP-conjugated secondary antibody and ECL detection.

**GST Pull-Down Assay**

Recombinant GST or GST-SIRT1 proteins (Sangon Biotech, China) were incubated with purified Flag-Nat10 protein for 4 hours, followed by the addition of Glutathione Sepharose 4B beads (GE Healthcare, USA). After washing, interactions were assessed via WB using anti-Flag antibodies to determine direct binding between SIRT1 and NAT10.

**RIP-qPCR Assay**

HEK293T cells (CL-0005, Procell, China) expressing Flag-*Nat10* were UV-crosslinked at 254 nm and lysed in hypotonic buffer (5 mM HEPES, pH 7.4; 85 mM KCl; 0.5% NP-40). Nuclei were pelleted and resuspended in a lysis buffer containing protease inhibitors (20 mM Tris-Cl, pH 8.0; 200 mM NaCl; 1 mM EDTA; 1 mM EGTA; 0.1% SDS; 1% NP-40; 0.5% deoxycholate), then sonicated. Lysates were pre-cleared with protein A agarose beads (GE Healthcare), and immunoprecipitation was performed at 4 °C for 2 hours using protein A beads pre-coated with anti-Flag M2 magnetic beads (M8823, Sigma-Aldrich, USA) or control rabbit IgG (ab172730, Abcam, UK). Complexes were washed once with IP buffer (20 mM Tris-Cl, pH 7.5; 150 mM NaCl; 0.1% NP-40), followed by two washes with high-salt IP buffer (400 mM KCl), each for 5 minutes. Bound RNA-protein complexes were eluted with Flag peptide (20 μg/100 μL, F3290, Sigma-Aldrich, USA) at 4 °C for 1 hour and de-crosslinked at 65 °C. RNA was extracted using TRIzol (15596026, Thermo Fisher, USA), treated with DNase I (M6101, Promega, USA), and reverse transcribed using random hexamers (N8080127, Thermo Fisher, USA) and MoMLV reverse transcriptase (10814360001, Roche, Switzerland). qPCR was performed to quantify the enrichment of *GABABR1* and *GABABR2* mRNAs.

**Statistical Analysis**

All data are presented as mean ± standard deviation (SD) from at least three independent experiments. Comparisons between the two groups were conducted using unpaired Student's t-tests. For multiple group comparisons, one-way ANOVA followed by Tukey's HSD post hoc test was employed. Nonparametric data were analyzed using the Mann–Whitney U test or Kruskal–Wallis H test, as appropriate. Statistical analyses were conducted using GraphPad Prism 9.5.0 (GraphPad Software, USA) and R version 4.2.1 (R Foundation for Statistical Computing). A two-tailed *p*-value < 0.05 was considered statistically significant.
